# Automatic target-seeking nanoparticle inhibiting orthotopic drug-resistant colon cancer and liver metastases via regulating cancer cell adhesion and proliferation

**DOI:** 10.1186/s12951-025-03422-x

**Published:** 2025-06-06

**Authors:** Shaobo Bai, Yang Sun, Miao Liu, Ying Cheng, Qifeng Ji, Bangle Zhang, Zhifu Yang, Siyuan Zhou, Daozhou Liu

**Affiliations:** 1 Department of Pharmaceutics, School of Pharmacy, Air Force Medical University, Xi’an, 710032 China; 2 Department of Pharmacy, The 942th Hospital of Joint Logistic Support Force of PLA, Yinchuan, 750001 China; 3 Key Laboratory of Gastrointestinal Pharmacology of Chinese Materia Medica of the State Administration of Traditional Chinese Medicine, Department of Pharmacology, School of Pharmacy, Air Force Medical University, Xi’an, 710032 China; 4 Department of Pharmacy, Xijing Hospital, Air Force Medical University, Xi’an, 710032 China; 5 Changle West Road 169, Shaanxi Province, Xi’an, 710032 Xi’an, China

**Keywords:** Drug-resistant colon cancer, Galectin-3, Cell adhesion, Paris saponin VII, Integrin αvβ3

## Abstract

Galectin-3 (Gal-3) plays an important role in adhesion and proliferation of cancer cells. The level of Gal-3 in blood and the expression of Gal-3 in colon cancer tissue are significantly increased in patient with colon cancer. The elevated Gal-3 promotes the migration and drug resistance of colon cancer. Therefore, Gal-3 is a promising target to inhibit the growth and metastases of cancer cells. Besides, integrin αvβ3, a receptor of Gal-3, is highly expressed in colon cancer cell and blood vessel in colon cancer tissue. In this paper, an automatic target-seeking nanoparticle (SP@MCaP) contained siGal-3 and paris saponin VII (PSVII) was prepared. In vivo, by automatically capturing Gal-3 in the blood circulation, SP@MCaP actively recognized cancer tissue vessel and drug-resistant colon cancer cells with elevated integrin αvβ3 expression, resulting in specifical accumulation in orthotopic drug-resistant colon cancer tissue. SP@MCaP diminished Gal-3 level in serum and orthotopic drug-resistant colon cancer tissue, and then suppressed the proliferation of drug-resistant colon cancer cells. Importantly, SP@MCaP reconstructed the adhesion of drug-resistant colon cancer cells and reversed the immunosuppressive microenvironment in orthotopic drug-resistant colon cancer tissue and liver tissue. Finally, under the synergistic effect of siGal-3 and PSVII, SP@MCaP successfully inhibited the growth of orthotopic drug-resistant colon cancer and its liver metastases. In a word, this paper explored a novel concept of the active co-delivery of siGal-3 and PSVII by modification of nanoparticle, which holds promise for targeted therapy in orthotopic drug-resistant colon cancer and its liver metastases.

**Graphical abstract:**

**Figure:**
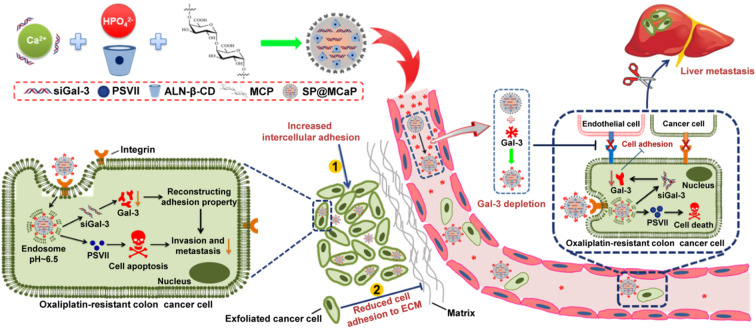
First, SP@MCaP automatically captured Gal-3 in the blood, actively recognized cancer tissue vessel and drug-resistant colon cancer cells with elevated integrin αvβ3 expression and specifically accumulated in orthotopic drug-resistant colon cancer tissue. Then, SP@MCaP successfully inhibited the growth of orthotopic drug-resistant colon cancer and its liver metastases by diminishing Gal-3 level in serum and orthotopic drug-resistant colon cancer tissue, suppressing the proliferation of drug-resistant colon cancer cells, reconstructing the adhesion of drug-resistant colon cancer cells and ameliorating the immunosuppressive microenvironment in orthotopic drug-resistant colon cancer tissue and liver tissue

**Supplementary Information:**

The online version contains supplementary material available at 10.1186/s12951-025-03422-x.

## Introduction

In recent years, with the improvement of living conditions, there has been a noticeable increase in both incidence and mortality of colorectal cancer (CRC). This makes it a pressing global health issue [1, 2]. According to the results of China’s cancer statistics in 2022, approximately 517,100 new cases of colorectal cancer have been reported with an estimated 240,000 deaths [3]. Among all types of cancers, colorectal cancer ranks second in terms of incidence and fourth in terms of mortality [4]. Presently, chemotherapy stands as a prevalent treatment for colon cancer. With the successive application of oxaliplatin and irinotecan, the chemotherapeutic effect against colon cancer has been significantly enhanced [5]. However, colon cancer is prone to develop resistance to commonly used chemotherapeutic drugs, which consequently leads to local recurrence and metastasis. The 5-year survival rate of colon cancer patients with metastasis is less than 15% [6, 7]. Therefore, novel approaches are urgently required to block the growth and metastasis of orthotopic drug-resistant colon cancer.

After the development of resistance to chemotherapy in colon cancer, the efficacy of chemotherapeutic agents diminishes or even ceases, resulting in rapid proliferation of colon cancer cells. This leads to an increased intratumoral growth pressure, and then compels resistant cancer cells to migrate towards areas of lower pressure. Thereby the invasion and metastasis of these malignant cells is facilitated. Therefore, to effectively inhibit the invasion and metastasis of drug-resistant colorectal cancer, it is essential to suppress the proliferation of drug-resistant colorectal cancer cells. In addition, uncontrolled adhesion of cancer cells is a crucial factor contributing to their invasion and metastasis. E-cadherin serves as a critical molecule in maintaining intercellular adhesion among cancer cells. Inhibition of E-cadherin expression decreases adhesion between these cells, resulting in the detachment of individual cancer cells from the primary tumor site. Furthermore, matrix metalloproteinases-9 (MMP-9) has been shown to degrade E-cadherin and diminish cell-to-cell adhesion, which facilitates the dissemination of cancer cells from cancer tissue.

Colon cancer cells and vascular endothelial cells in colon cancer tissues exhibit an elevated expression of integrin αvβ3 [8], whereas inactivated vascular endothelial cells and majority of normal cells display less expression of integrin αvβ3. Galectin-3 (Gal-3), a glycoprotein, is a ligand of integrin αvβ3 [9]. Gal-3 is synthesized within cancer cells and can be secreted extracellularly. Gal-3 plays a crucial role in facilitating the dissemination of cancer cells within the body. By binding with integrin αvβ3, Gal-3 closely connects cancer cells with fibulin and laminin in the surrounding matrix. This provides fulcrum for shed cancer cells, and then promotes metastasis of cancer cells [10, 11]. Gal-3 also attenuates adhesion between cancer cells in cancer tissue tthrough reducing the expression of E-cadherin and increasing the expression of N-cadherin in cancer cells, thereby promoting their separation from each other. This facilitates cancer cells escape from the primary site [12, 13]. Furthermore, high level of Gal-3 induces circulating cancer cells to expose themselves to E-cadherin and E-selectin, enabling circulating cancer cells to aggregate. Then cancer thrombus is formed to evade anoikis and elimination by the blood immune system [14]. Additionally, intracellular Gal-3 inhibits cytochrome C release by binding to Bcl-2 [15], consequently impeding tumor cell apoptosis [16, 17]. Clinical studies have revealed that the higher the level of Gal-3 in serum and cancer tissue, the higher the incidence of colon cancer metastasis [18–20]. Therefore, Gal-3 is considered as a novel target to inhibit both growth and metastasis of colon cancer.

Paris saponin VII (PSVII) possesses the ability to inhibit the growth of drug-resistant colon cancer [21, 22]. However, it does not exhibit inhibitory effects on the migration of cancer cells. Besides, PSVII can’t block the adhesion between cancer cells in the blood circulation and their attachment to vascular endothelium at the metastatic sites. As a result, PSVII has no obvious effect on thwarting the metastasis of drug-resistant colon cancer. Therefore, the simultaneous delivery of siGal-3 and PSVII to drug-resistant colon cancer cells can synergistically suppress the growth and metastasis of drug-resistant colon cancer by remodeling cell adhesion and promoting apoptosis of drug-resistant colon cancer cells.

**Scheme 1:**
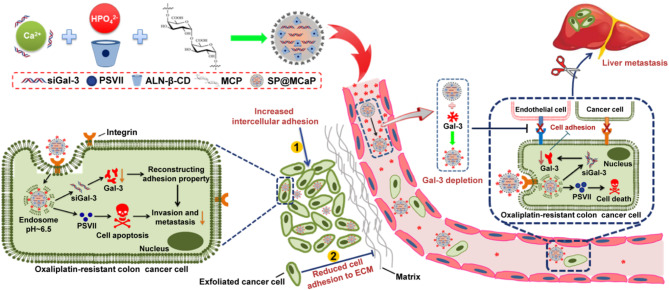
The mechanism of an automatic target-seeking nanoparticle contained siGal-3 and PSVII

Calcium phosphate nanoparticles are exceptional delivery vectors for siRNA due to their superior transfection efficiency, excellent biocompatibility and biodegradability [23, 24]. Nevertheless, calcium phosphate nanoparticles tend to aggregate during preparation and lack active targeting to colon cancer [25]. Modified citrus pectin (MCP) contains carboxyl group, and it can complex Ca^2+^. Thus MCP is used as a stabilizer for calcium phosphate nanoparticles [21]. In addition, MCP serves as a natural ligand of Gal-3 [26, 27]. Therefore, MCP-reinforced calcium phosphate nanoparticles can actively capture Gal-3 in the blood circulation. Because αvβ3 is highly expressed in vascular endothelial cells in colon cancer tissue and drug-resistant colon cancer cells, Gal-3 modified nanoparticle can actively accumulate in tumor tissue through the interaction between Gal-3 and integrin αvβ3. In this study, by using PSVII loaded alendronic acid-β-cyclodextrin inclusion complex (PSVII@ALN-β-CD) and MCP as stabilizers, calcium phosphate nanoparticles co-loaded with siGal-3 and PSVII (SP@MCaP) were prepared. SP@MCaP effectively delivered siGal-3 and PSVII to drug-resistant colon cancer cells by actively capturing Gal-3 in blood as targeting ligand. Subsequently, SP@MCaP inhibited the growth and metastasis of drug-resistant colon cancer by suppressing proliferation and remodeling adhesion of drug-resistant colon cancer cells (Scheme 1).

## Materials and methods

### Materials

Modified citrus pectin (MCP) was purchased from Centrax International (USA). PSVII was obtained from Mansite (Chengdu, China). Integrin αvβ3 antibody was purchased from NOVUS (Colorado, USA), and all other antibody were bought from Abcam (Cambridge, England). Integrin αvβ3 was purchased from R&D (Minnesota, USA). Enzyme linked immunosorbent assay (ELISA) kits for TNF-α, TGF-β, IFN-γ, Gal-3 and IL-10 were obtained from Jianglai Biotechnology (Shanghai, China). siGal-3 (5’-CUCGCAUGCUGAUAACAAUTT-3’,5’-AUUGUUAUCAGCAUGCGAGTT-3’) and cyanine5 labeled siGal-3 were bought from GenePharma (Shanghai, China). HCT116 cell and NCM460 cell were bought from ATCC (Maryland, USA). Oxaliplatin-resistant HCT116 cell (HCT116/L cell) was obtained from Zhewen Biotechnology (Shanghai, China). Nude mice were obtained from the Experimental Animal Center of Air Force Medical University (Xi’an, China).

### Expression of integrin αvβ3 in HCT116/L cells and activated HUVEC cells

NCM460 cells, HCT116 cells and HCT116/L cells at logarithmic growth stage were seeded into 6-well cell culture plates (1 × 10^6^ cells/well) and incubated for 24 h. The expression of integrin αvβ3 was detected by western blot. Additionally, the distribution of integrin αvβ3 in NCM460 cells, HCT116 cells and HCT116/L cells was observed by fluorescence immunoassay.

HCT116/L cells at logarithmic growth stage were seeded into a 6-well cell culture plate (1 × 10^6^ cells/well) and cultured for 24 h. Subsequently, the cell culture medium was collected. HUVEC cells at logarithmic growth stage were seeded into a 6-well cell culture plate (1 × 10^6^ cells/well) and cultured for 24 h. The cell culture medium was then replaced with the collected culture medium from HCT116/L cells and cultured for 48 h. Ultimately, the protein of HUVEC cells was extracted, and the expression of integrin αvβ3 in HUVEC cells was investigated by western blot.

### Preparation and characterization of G-SP@MCaP

Initially, the siGal-3 solution (20 µM), CaCl_2_ solution (125 mM), Na_2_HPO_4_ solution (10 mM) and MCP solution (0.5 mg/mL) were individually prepared using DEPC water. Subsequently, 5 µL of siGal-3 solution and 5 µL of CaCl_2_ solution were thoroughly mixed in an enzyme-free centrifuge tube. Then, 5 µL of Na_2_HPO_4_ solution containing PSVII@ALN-β-CD (1, 2.5, 5, 10, 15, 20 mg/mL) was added and left for 30 min. Next, 5 µL of MCP solution was added to the above mixture and thoroughly mixed before being left for another 15 min. Finally, the solution was centrifuged for 15 min (13680×g, 4℃), and the resulting precipitation was calcium phosphate nanoparticles co-loaded with siGal-3 and PSVII (SP@MCaP). The residue of siGal-3 in the supernatant was investigated by agarose gel electrophoresis to screen for optimal SP@MCaP prescription based on residual siGal-3 level [28]. Appropriate amount of SP@MCaP was dispersed with deionized water, then 20 ng of Gal-3 protein was added and incubated at 37 ℃ for 1 h. The solution was centrifuged (13680×g, 4 ℃) to separate precipitation. After washing the precipitation with deionized water for 3 times, the precipitation was SP@MCaP absorbed with Gal-3 protein, namely G-SP@MCaP. Calcium phosphate nanoparticle without MCP (SP@CaP) was prepared by the same method as above. The particle size, PDI, Zeta potential and stability of SP@MCaP and G-SP@MCaP were investigated by nanometer laser particle size analyzer (Malvern ZEN 3600, England). Simultaneously, SP@MCaP was prepared with F-labeled siGal-3 and S-labeled PSVII, and the morphology and chemical element of SP@MCaP were observed by transmission electron microscope mapping analysis (TEM, JEM-F200, Japan). Gal-3 in G-SP@MCaP was characterized by SDS-PAGE gel electrophoresis and ELISA [29]. The protective effect of G-SP@MCaP on siGal-3, as well as the release of siGal-3 from G-SP@MCaP were investigated by agar-gel electrophoresis. Furthermore, the corrosion of G-SP@MCaP in deionized water with pH 5.0 was observed by TEM.

The drug-loading of PSVII in SP@MCaP and the release of PSVII from G-SP@MCaP were detected with ultra-high performance liquid chromatograph (UPLC, Waters, USA). The equilibrium dissociation constant (Kd) between SP@MCaP and Gal-3, as well as G-SP@MCaP and integrin αvβ3 were determined by microscale thermophoresis (MST, Nano Temper, Germany) [30]. ELISA kit was utilized to detect the adsorption of IgG, IgM, C3b and C4b by SP@CaP and SP@MCaP in mice serum. The hemolysis rate of G-SP@MCaP in 2% red blood cell suspension of rat was determined by spectrophotometry at 414 nm [31], and the effect of G-SP@MCaP on the integrality of red blood cell was observed by scanning electron microscope (SEM, Hitachi, Japan).

### The trafficking of G-SP@MCaP in HCT116/L cell

HCT116/L cells at logarithmic growth stage were seeded into a 24-well cell culture plate (1 × 10^5^ cells/well) with small glass discs and cultured for 24 h. Subsequently, Cy5-labeled G-SP@MCaP was introduced into the cell culture medium, and the cells were cultured for 4 h. Following fixation with 4% paraformaldehyde solution, the integrin αvβ3 on HCT116/L cells were labelled with αvβ3 antibody (Alexa Fluor^®^ 488) and the nucleus were labelled with DAPI. The colocalization of integrin αvβ3 and G-SP@MCaP in HCT116/L cells was observed by laser scanning confocal microscopy (LSCM, Olympus, Japan).

HCT116/L cells at logarithmic growth stage were seeded into a 24-well cell culture plate (1 × 10^5^ cells/well) with small glass discs and cultured for 24 h. Cy5-labeled G-SP@MCaP was added into the cell culture medium, and cells were cultured for 2 h and 4 h. After being fixed with 4% paraformaldehyde solution, the cells were treated with 0.1% Triton X-100 solution for 10 min. The lysosomes of HCT116/L cells were labelled with LAMP1 antibody (Alexa Fluor^®^ 488) and the nucleus were labelled with DAPI. The distribution of G-SP@MCaP in lysosomes was observed by LSCM.

### The uptake of G-SP@MCaP by HCT116/L cell

NCM460 cells, RAW264.7 cells and HCT116/L cells at logarithmic growth stage were seeded into 24-well cell culture plates (1 × 10^5^ cells/well) with small glass discs and cultured for 24 h. The small glass discs with different cells were placed into the same hole of the 6-well cell culture plate, and serum-free RPMI medium containing SP@MCaP (Cy5 labeled) and G-SP@MCaP (Cy5 labeled) was added. The cells were cultured for 0.5 h and 4 h. (1) After the cells were respectively collected, the uptake of G-SP@MCaP by NCM460 cells, RAW264.7 cells and HCT116/L cells was investigated by flow cytometry (Beckman Coulter, USA). (2) Following removal of the culture medium, the cells were respectively fixed with 4% paraformaldehyde solution. Finally, the cells were incubated with DAPI solution, and the uptake of G-SP@MCaP by NCM460 cells, RAW264.7 cells and HCT116/L cells was observed by LSCM.

HCT116/L cells at logarithmic growth stage were seeded into a 24-well cell culture plate (1 × 10^5^ cells/well) with small glass discs and cultured for 24 h. After removing the cell culture medium, the cells were incubated with serum-free RPMI medium containing methyl-β-cyclodextrin (5 µg/mL), colchicine (800 µg/mL), chlorpromazine (10 µg/mL), c (RGDfK) cyclopeptide (2 nM), Gal-3 (0.1 µg/mL), and 2-deoxyglucose (950 µg/mL) for 1 h, respectively. Additionally, the cells in the low temperature group were incubated in the refrigerator at 4℃ for 1 h. Subsequently, Cy5-labeled G-SP@MCaP was added into cell culture medium, and cells were cultured for 4 h. (1) After the cells were collected, the uptake of G-SP@MCaP by HCT116/L cells was investigated by flow cytometry. (2) Following removal of the culture medium, the cells were fixed with 4% paraformaldehyde solution. Finally, the cells were incubated with DAPI solution, and the uptake of G-SP@MCaP by HCT116/L cells was observed by LSCM.

### Toxicity of G-SP@MCaP on HCT116/L in vitro

HCT116/L cells at logarithmic growth stage were seeded into 96-well cell culture plates (1 × 10^4^ cells/well) and cultured for 24 h. Subsequently, the culture medium was replaced with serum-free RPMI medium containing S@MCaP (calcium phosphate nanoparticles containing only siGal-3), P@MCaP (calcium phosphate nanoparticles containing only PSVII), SP@MCaP and G-SP@MCaP, and the cells were cultured for 48 h. The serum free culture medium with no drug served as the control. Following this, 20 µL of MTT solution (5 mg/mL) was added to each well, the cells were further cultured for 4 h. After removal of the culture medium, 150 µL of dimethyl sulfoxide solution was added to each well, and the absorbance value of each well was determined at 490 nm by using Molecular device (CMax Plus, China). Ultimately, the cell survival rate was calculated.

HCT116/L cells at logarithmic growth stage were seeded into a 6-well cell culture plate (1 × 10^6^ cells/well) and cultured for 24 h. Subsequently, the culture medium was replaced with serum-free RPMI medium containing S@MCaP, P@MCaP, SP@MCaP and G-SP@MCaP, and cells were further cultured for 48 h. The concentrations of siGal-3 and PSVII were 3 µg/mL, while the control group received serum-free culture medium. Following collection, the cells were stained with a live and dead cell staining kit and observed under a fluorescence microscope (Nikon, TS2R, Japan). Finally, the ratio of dead/alive cells was calculated.

HCT116/L cells at logarithmic growth stage were seeded into a 6-well cell culture plate (5 × 10^2^ cells/well) and cultured for 24 h. The medium was replaced with serum-free RPMI medium containing S@MCaP, P@MCaP, SP@MCaP and G-SP@MCaP, and cultured for 24 h. The concentrations of siGal-3 and PSVII were 3 µg/mL, while the control group received serum-free medium. Subsequently, the culture medium was switched to RPMI complete culture medium with continuous cultivation. The culture medium was replaced with fresh medium every 2 days until spherical cell clones emerged. Following fixation with 4% polyformaldehyde solution, the cell clones were stained with crystal violet and photographed for observation.

### Migration and invasion of HCT116/L cells

HCT116/L cells at logarithmic growth stage were seeded into transwell donor chamber (6 × 10^4^ cells/well). For invasion experiment, the transwell donor chamber was precoated with matrix gel. Subsequently, 500 µL of RPMI complete culture medium was added into the recipient chamber. After 4 h, the medium in donor chamber was replaced with S@MCaP, P@MCaP, SP@MCaP and G-SP@MCaP, and cells were cultured for an additional 24 h. The concentrations of siGal-3 and PSVII were 3 µg/mL, while serum-free medium served as the control. After gently swabbing the interior of the donor chamber with a cotton swab, the cells were fixed with 4% paraformaldehyde solution and subsequently stained with 0.1% crystal violet solution. The cell migration and invasion were then observed by inverted microscope. Finally, each well was treated with 33% glacial acetic acid to dissolve crystal violet, and the absorbance value at 570 nm was measured by Molecular device to calculate relative cell mobility based on the ratio of absorbance between drug treatment group and control group.

HCT116/L cells at logarithmic growth stage were seeded into a 6-well cell culture plate (1 × 10^6^ cells/well) and cultured for 24 h. Subsequently, the cell culture medium was replaced with serum-free RPMI medium containing NP@MCaP (siNC/PSVII@MCaP), SP@MCaP, G-SP@MCaP and S@Lipo2000, and the cells were further cultured for 48 h. The contents of siGal-3 and siNC were 300 pmol, while serum-free medium served as the control. Following removal of the culture medium, the cell proteins were extracted. Finally, the expression of Gal-3 was investigated by western blot.

HCT116/L cells at logarithmic growth stage were seeded into a 6-well cell culture plate (1 × 10^6^ cells/well) and cultured for 24 h. Subsequently, the cell culture medium was replaced with serum-free RPMI medium containing S@MCaP, P@MCaP, SP@MCaP and G-SP@MCaP, and the cells were further cultured for 48 h. The concentrations of siGal-3 and PSVII were 3 µg/mL, while serum-free medium served as the control. Following removal of the culture medium, the cell proteins were extracted. Finally, the expressions of apoptosis-related proteins, invasion-related proteins and motion-related proteins were investigated by western blot.

### Adhesive characteristic of HCT116/L cells

HCT116/L cells at logarithmic growth stage were seeded into a 24-well cell culture plate (5 × 10^4^ cells/well) with small glass discs and cultured for 24 h. Subsequently, the culture medium was replaced with serum-free RPMI medium containing S@MCaP, P@MCaP, SP@MCaP and G-SP@MCaP, and the cells were further cultured for 24 h. The concentrations of siGal-3 and PSVII were 3 µg/mL, while serum-free medium served as the control. Following fixation with 2.5% glutaraldehyde solution, the cells underwent dehydration with ethanol. Ultimately, the cell morphology and pseudopodia were observed by SEM.

GFP-HCT116/L cells at logarithmic growth stage were seeded into a 24-well cell culture plate (5 × 10^5^ cells/well) and cultured for 24 h. Subsequently, the culture medium was replaced with serum-free RPMI medium containing S@MCaP, P@MCaP, SP@MCaP and G-SP@MCaP, and the cells were further cultured for 24 h. The concentrations of siGal-3 and PSVII were 3 µg/mL, while serum-free medium served as the control. (1) GFP-HCT116/L cells (3 × 10^5^) were harvested and incubated with HCT116/L cells which was labeled cytoplasmic red fluorescent probes. After incubation for 1 h, the non-adherent GFP-HCT116/L cells were gently washed off with PBS solution. The adhesion between GFP-HCT116/L and HCT116/L cells was observed under a fluorescence microscope. (2) GFP-HCT116/L cells (3 × 10^5^) were seeded into a matrix-coated 96-well plate for 1 h incubation. The non-adherent GFP-HCT116/L cells were subsequently washed off with PBS, while the adherent cells were stained with 0.1% crystal violet solution. Finally, each well was treated with 33% glacial acetic acid to dissolve crystal violet, and the absorbance value at 570 nm was measured by Molecular device. The relative adhesion rate of cells was calculated by comparing their absorbance to that of the control group.

The GFP-HCT116/L cells at logarithmic growth stage were seeded into a 24-well cell culture plate (5 × 10^5^ cells/well) for 24 h. The culture medium was then replaced with serum-free RPMI medium containing S@MCaP, P@MCaP, SP@MCaP and G-SP@MCaP, and cells were cultured for 24 h. The concentrations of siGal-3 and PSVII were 3 µg/mL, while the serum-free medium served as the control. HUVEC cells at logarithmic growth stage were seeded into a 24-well cell culture plate (5 × 10^5^ cells/well) and cultured for 24 h. Subsequently, the GFP-HCT116/L cell culture medium was collected and centrifuged (13680×g, 4℃). The resulting supernatant was added into the HUVEC cells and cultured for 24 h. Following this, the HUVEC cell culture medium was collected. GFP-HCT116/L cells at logarithmic growth stage were seeded into a 24-well cell culture plate (5 × 10^5^ cells/well) and cultured for 24 h. The cell culture medium was then replaced with the above HUVEC cell culture medium, and cells were cultured for 24 h. The GFP-HCT116/L cells were then prepared into cell suspension. HUVEC cells at logarithmic growth stage were seeded in 24-well cell culture plate (3 × 10^5^ cells/well) and cultured for 24 h. Subsequently, 500 µL of cytoplasmic red fluorescent probe solution (1 µM) was added and cells were cultured for 20 min. Following this, the GFP-HCT116/L cell suspension (3 × 10^5^) was added into HUVEC cells and cultured for 1 h. The non-adherent GFP-HCT116/L cells were washed off gently with PBS solution, and the adhesion between HCT116/L cells and HUVEC cells was observed by fluorescence microscope.

HCT116/L cells at logarithmic growth stage were seeded into a 6-well cell culture plate (1 × 10^6^ cells/well) and cultured for 24 h. Subsequently, the cell culture medium was replaced with serum-free RPMI medium containing S@MCaP, P@MCaP, SP@MCaP and G-SP@MCaP, and cells were cultured for 24 h. The concentrations of siGal-3 and PSVII were 3 µg/mL, while the serum-free medium served as the control. The culture medium was then centrifuged (13680×g, 4 ℃), and the resulting supernatant was collected. HUVEC cells at logarithmic growth stage were seeded into a 6-well cell culture plate (1 × 10^6^ cells/well) and cultured for 24 h. The cell culture medium was then replaced with the collected supernatant from HCT116/L cells and cultured for 24 h. Ultimately, the protein of HUVEC cells was extracted, and the expression of ICAM-1 in HUVEC cells was investigated by western blot.

HCT116/L cells were suspended in serum-free RPMI medium containing S@MCaP, P@MCaP, SP@MCaP and G-SP@MCaP (5 × 10^3^ /mL). The concentrations of siGal-3 and PSVII were 3 µg/mL, while the serum-free medium served as the control. 10 mL of HCT116/L cell suspension was added into a cell culture flask (T25) and cultured under oscillation for 6 h. The formation of HCT116/L cell aggregates was observed with an inverted microscope.

HUVEC cells were suspended in serum-free RPMI medium containing S@MCaP, P@MCaP, SP@MCaP and G-SP@MCaP (4 × 10^5^ cells/mL). The concentrations of siGal-3 and PSVII were 3 µg/mL, while the serum-free medium served as the control. 50 µL of HUVEC cells were added to a 96-well cell culture plate precoated with matrix glue and cultured for 24 h. The formation of cell tubules was observed and counted by inverted microscope.

### Biodistribution of SP@MCaP in mice with orthotopic drug-resistant colon cancer

HCT116/L-luc cells at logarithmic growth stage were resuspended with serum-free RPMI medium (5 × 10^7^ cells/mL). HCT116/L-luc cell suspension (200 µL) was injected subcutaneously into the back of nude mice. Once the diameter of the subcutaneous cancer reached approximately 1.5 cm, the subcutaneous cancer tissue was collected and cut into 3 × 3 × 1 mm^3^ squares. Subsequently, in another batch of nude mice, anesthesia was administered before making a small incision in the middle of the abdomen. Cancer tissue was implanted in the colon about 1 cm away from the cecum and fixed with biogel [32]. Then the incision was sutured. Eventually, the growth of drug-resistant colon cancer in nude mice was observed by in vivo imaging (caliper life sciences, USA).

Nude mice with orthotopic drug-resistant colon cancer were given Cy5-labeled siGal-3, Cy5-labeled SP@CaP, and Cy5-labeled SP@MCaP via the tail vein (0.3 mg/kg, measured by siGal-3-Cy5 content). Blood samples were collected at 12 h and 24 h post-administration, along with brain, heart, liver, spleen, lung, kidney and drug-resistant colon cancer tissues were collected. The fluorescence intensity in plasma was measured by fluorescence spectrophotometer, and in vivo imaging was utilized to observe the fluorescence intensity in the organs and tissues of nude mice. Subsequently, adjacent normal colon tissue and colon cancer tissue were harvested and fixed with 4% paraformaldehyde solution. The tissues were processed into paraffin sections and then stained with DAPI. The fluorescence distribution in adjacent normal colon tissues and colon cancer tissues was observed by LSCM.

### Inhibition of SP@MCaP on the growth of orthotopic drug-resistant colon cancer

After implantation of colon cancer tissue, the nude mice were administered with normal saline, N@MCaP (calcium phosphate nanoparticles loaded with negative control siRNA), S@MCaP (calcium phosphate nanoparticles loaded with siGal-3), P@MCaP (calcium phosphate nanoparticles loaded with PSVII), SP@CaP (calcium phosphate nanoparticles loaded with siGal-3 and PSVII without MCP modification), S@MCaP + P@MCaP, and SP@MCaP through the tail vein on the day of 7th, 11th, 15th and 19th. The dosage of siGal-3 and PSVII in the nanoparticles was 1 mg/kg. Additionally, on the day of 5th, 14th and 22th after the transplantation of cancer tissue pieces, the nude mice were given D-luciferin potassium salt solution (150 mg/kg) by intraperitoneal injection to observe their fluorescence intensity by in vivo imaging. During the treatment, the body weight of nude mice was recorded at an interval of 1 day. On the third day after the last administration, mice were sacrificed. Simultaneously, colon tissue was collected and photographed. Colon cancer tissues were gathered and weighed. After fixing with 4% paraformaldehyde solution, colon cancer tissues were prepared into paraffin sections and then stained with TUNEL, Ki67 and *H&E*. Additionally, the expressions of Gal-3, CD31 (marker of blood vessel) and CD3^−^/NK1.1^+^ (marker of NK cell) in colon cancer tissues were investigated by immunofluorescence staining. The levels of IL-10, TGF-β, TNF-α and IFN-γ in colon cancer tissues were detected by ELISA kit. Finally, the expression of Gal-3, apoptosis-associated proteins (Bax, Bcl-2 and Cleaved caspase-3), invasion-related proteins (E-cadherin, CD44, N-cadherin and MMP-9) and motion-related proteins (RhoA and Cdc42) were analyzed by western blot.

### Suppression of SP@MCaP on liver metastasis of colon cancer

HCT116/L-luc cells at logarithmic growth stage were resuspended with serum-free RPMI medium (5 × 10^7^ cells/mL). After anesthesia, the abdomen of nude mice was incised along the left costal margin, and the spleen was exteriorized. 200 µL of HCT116/L-luc cell suspension was slowly injected into spleen through the lower end of spleen to the middle [33]. After applying cotton swab to stop bleeding, the spleen was returned to the abdominal cavity and the incision was closed. Liver metastases of drug-resistant colon cancer in nude mice were observed using in vivo imaging.

After inoculation of HCT116/L-luc cells in the spleen, normal saline, S@MCaP, P@MCaP, SP@CaP and SP@MCaP were administered to nude mice via the tail vein injection on the day of 0th, 4th, 8th and 12th. Additionally, D-luciferin potassium salt solution (150 mg/kg) was injected intraperitoneally on the 4th and 15th days after cell inoculation and the fluorescence intensity in abdomen was observed by in vivo imager. On the third day after the last administration, nude mice were sacrificed, and serum was isolated. Simultaneously, liver tissue was collected and photographed. Subsequently, the liver tissues were fixed with 4% paraformaldehyde solution before preparation into paraffin sections for *H&E* staining. The proportion of metastatic cancer area was calculated by ImageJ software. Serum Gal-3 levels in nude mice with liver metastasis of colon cancer were detected by ELISA kit. Finally, the expressions of Gal-3, CD3^−^/NK1.1^+^ (marker of NK cell) and CD31/ICAM-1 in liver of nude mice with liver metastasis of colon cancer were detected by immunofluorescence staining.

### Preliminary safety of SP@MCaP in vivo

Nude mice were administered with normal saline, N@MCaP, S@MCaP, P@MCaP, SP@CaP, S@MCaP + P@MCaP, and SP@MCaP by the tail vein injection. The dosages of siGal-3 and PSVII in nanoparticles were 1 mg/kg. Drugs were given once every 3 days, and 4 doses in total. On the 3rd day after the final dosing, serum samples were collected to detect the alanine aminotransferase (ALT) activity, aspartate aminotransferase (AST) activity, lactate dehydrogenase (LDH) activity, urea nitrogen (BUN) content and creatinine (CREA) content. Simultaneously, the nude mice were sacrificed and the brain, heart, liver, spleen, lung and kidney tissue were gathered for *H&E* staining [34].

### Statistical analysis

The experimental data were presented as mean ± SD and statistically analyzed by SPSS 16.0 software. Comparison between two groups was performed by t-test, and comparison between multiple groups was performed by one-way analysis of variance. *P* < 0.05 indicated that the difference was statistically significant.

## Results and discussion

### SP@MCaP actively captured Gal-3 in the serum and improved stability of siGal-3 in vitro

**Fig. 1:**
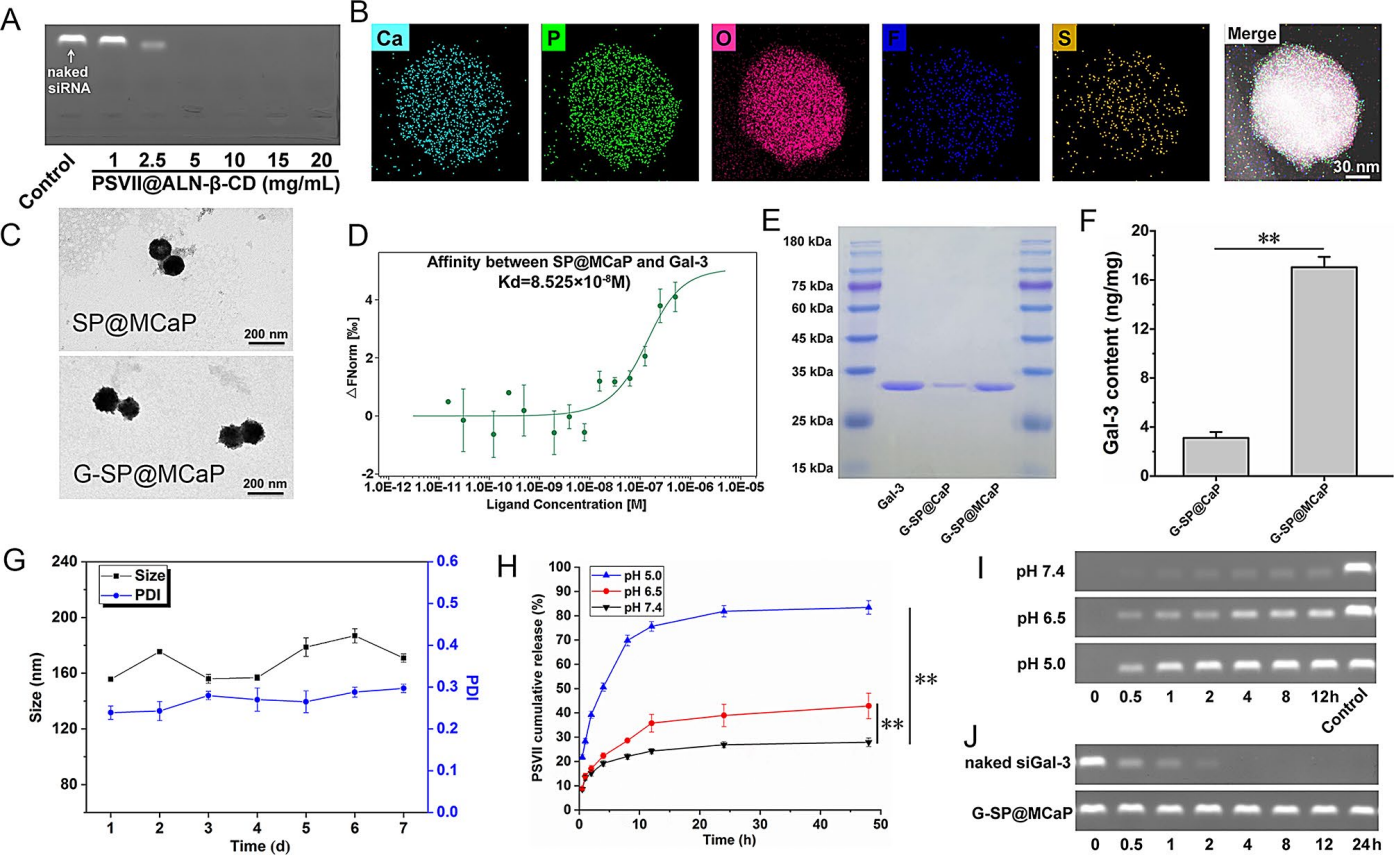
Characteristics of G-SP@MCaP. (**A**) The effect of PSVII@ALN-β-CD on siGal-3 loaded by calcium phosphate nanoparticle. (**B**) The TEM mapping element analysis of SP@MCaP. (**C**) TEM images of SP@MCaP and G-SP@MCaP. (**D**) The affinity between SP@MCaP and Gal-3 detected by microscale thermophoresis. (**E**) The capture of Gal-3 by SP@MCaP in PBS. (**F**) The capture of Gal-3 by SP@MCaP in serum of nude mice with orthotopic drug-resistant colon cancer. (**G**) Changes in particle size of G-SP@MCaP in normal saline solution. (**H**) Release of PSVII from G-SP@MCaP under different pH medium. (**I**) Release of siGal-3 from G-SP@MCaP under different pH medium. (**J**) The stability of siGal-3 in G-SP@MCaP. *n* = 3, mean ± SD, ^**^P < 0.01

Calcium phosphate nanoparticle belongs to a metastable state, it is prone to agglomeration and transition to a crystalline form. Compounds containing carboxyl group and phosphoric acid have the ability to complex with Ca^2+^ [35], thereby playing a crucial role in stabilizing calcium phosphate nanoparticles [36]. Therefore, in order to prepare stable calcium phosphate hybrid nanoparticles for loading siGal-3 and PSVII, carboxyl-abundant MCP and PSVII loaded alendronic acid-β-cyclodextrin inclusion complex (PSVII@ALN-β-CD) were used as stabilizers. When the concentration of PSVII@ALN-β-CD exceeded 5 mg/ml, no obvious siGal-3 bands were observed, indicating complete loading of siGal-3 in SP@MCaP (Fig. 1A). Additionally, after marking siGal-3 with F element and PSVII with S element, TEM mapping element analysis of SP@MCaP was performed. Strong signals of Ca, P, O, F, and S elements were found in SP@MCaP (Fig. 1B), confirming successful encapsulation of siGal-3 and PSVII within SP@MCaP. The particle size and zeta potential of SP@MCaP were 142 ± 4 nm and − 14.9 ± 1.5 mV, respectively (Fig.S1). Furthermore, SP@MCaP exhibited a spherical appearance with a drug loading of (3.06 ± 0.25)% for PSVII (Fig. 1C).

The results of microscale thermophoresis (MST) experiments demonstrated that SP@MCaP exhibited a high affinity with Gal-3 protein, with a Kd value of 8.525 × 10^− 8^ M (Fig. 1D). This indicated that SP@MCaP had the capability to automatically capture Gal-3. In PBS containing Gal-3, the adsorption capacity of SP@MCaP for Gal-3 was significantly superior to that of SP@CaP (Fig. 1E). Furthermore, in the serum of nude mice with orthotopic drug-resistant colon cancer, SP@MCaP adsorbed approximately 5.5 times more Gal-3 as compared to SP@CaP (Fig. 1F). These results highlighted the crucial role played by MCP in absorbing Gal-3, and demonstrated that SP@MCaP could automatically trap Gal-3 as a targeting ligand to form G-SP@MCaP in the blood circulation. G-SP@MCaP displayed a particle size of 151 ± 3 nm and a zeta potential of -4.3 ± 0.6 mV (Fig.S1). The appearance of G-SP@MCaP was spherical with uniform particle size distribution (Fig. 1C). Additionally, G-SP@MCaP was stable in normal saline solution (Fig. 1G). However, when incubation with pH 5.0 medium, the number and particle size of G-SP@MCaP gradually decreased (Fig.S2), indicating that G-SP@MCaP gradually dissolved in acidic environment. Therefore, as the pH of release medium decreased, there was a significant acceleration in the release of siGal-3 and PSVII from G-SP@MCaP (Fig. 1H-I). This indicated that G-SP@MCaP kept stability in neutral environment and had distinct pH-responsive drug release characteristics.

The degradation of siRNA by nuclease will inevitably reduce its silencing effect in vivo [37, 38]. After incubating with 10% serum for 24 h, siGal-3 was scarcely degraded in G-SP@MCaP, suggesting G-SP@MCaP enhanced the stability of siGal-3 in serum (Fig. 1J).

### SP@MCaP was safe in vitro and in vivo

PSVII can form insoluble complexes with cholesterol in red blood cell membrane, thereby increasing the osmotic pressure of red blood cells. This leads to the hemolysis [39]. As compared with free PSVII, G-SP@MCaP significantly reduced the hemolysis effect of PSVII (Fig.S3). *H&E* staining results revealed no significant abnormality in organ morphology of nude mice in SP@MCaP groups. Additionally, ALT activity, AST activity, LDH activity, BUN content and CREA content were all within the normal range after administration of SP@MCaP to normal nude mice. The above data suggested that SP@MCaP did not induce damage to liver, kidney and myocardium in normal nude mice at the therapeutic dose (Fig.S4).

### The capture of Gal-3 improved the targeting ability of SP@MCaP to orthotopic drug-resistant colon cancer

**Fig. 2:**
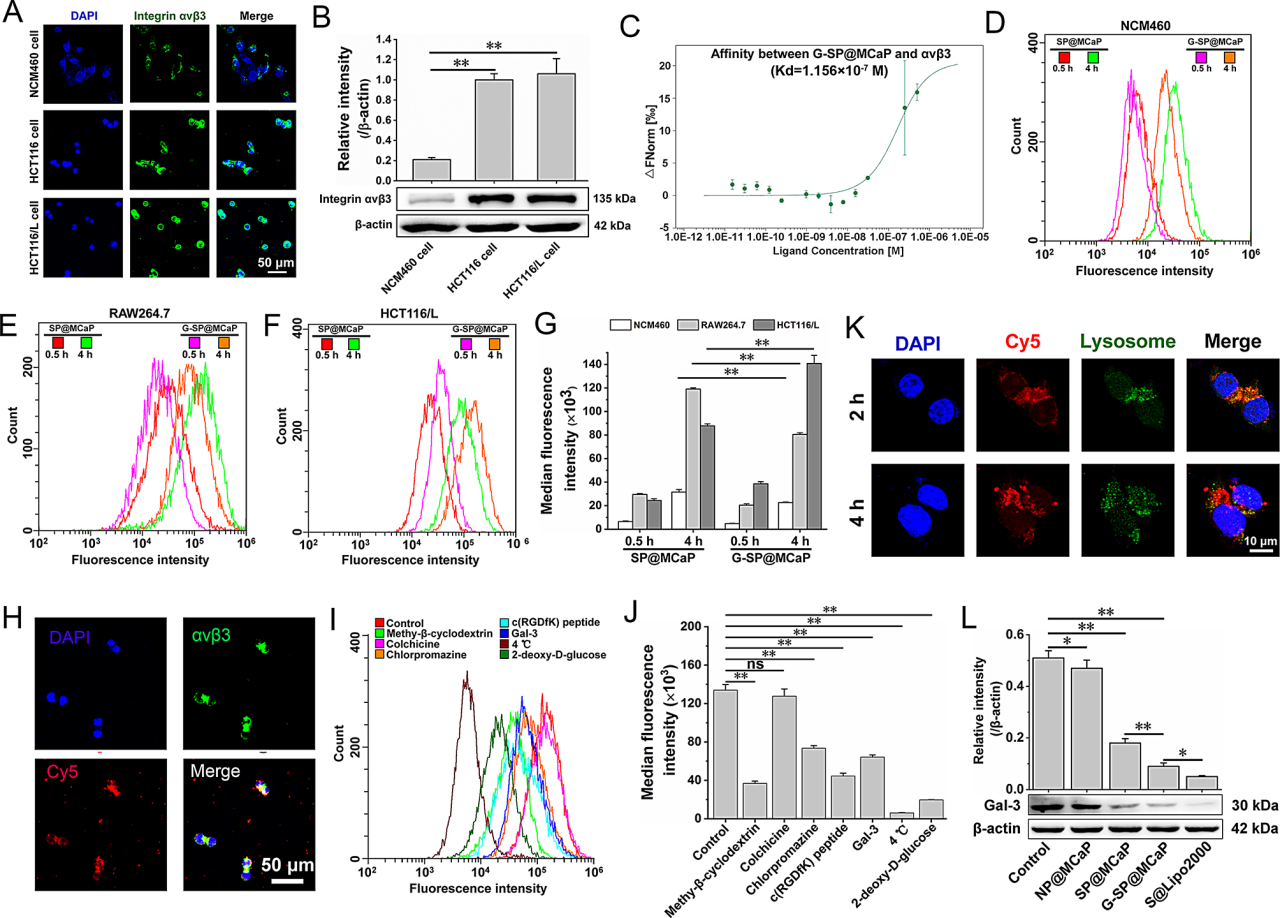
Uptake of G-SP@MCaP by HCT116/L cells and its mechanism. (**A-B**) Expression of integrin αvβ3 in normal colon epithelial cells (NCM460 cells) and colon cancer cells (HCT116 cells and HCT116/L cells). (**C**) The affinity between G-SP@MCaP and integrin αvβ3 detected by microscale thermophoresis. (**D-G**) Uptake of G-SP@MCaP by co-cultured NCM460 cells, RAW264.7 cells and HCT116/L cells. (**H**) Typical LSCM picture of colocalization of G-SP@MCaP and integrin αvβ3 in HCT116/L cells. (**I-J**) Mechanism of the uptake of G-SP@MCaP by HCT116/L cells. (**K**) Typical LSCM picture of siGal-3 escape from the lysosomes of HCT116/L cells. (**L**) The silencing effect of G-SP@MCaP on the expression of Gal-3 in HCT116/L cells. *n* = 3, mean ± SD, ^*^P < 0.05, ^**^P < 0.01, ns: no significant difference

The expression of integrin αvβ3 in normal colon epithelial cells (NCM460 cells) and colon cancer cells (HCT116 cells and HCT116/L cells) was investigated by immunofluorescence staining and western blot. The results indicated that integrin αvβ3 was highly expressed in both HCT116 and HCT116/L cells compared to NCM460 cells (Fig. 2A-B). Additionally, the affinity between G-SP@MCaP and integrin αvβ3 was investigated by MST experiments, and the Kd value was 1.156 × 10^− 7^ M (Fig. 2C), suggesting that G-SP@MCaP had a high affinity with integrin αvβ3. Consequently, G-SP@MCaP had the potential to actively target to drug-resistant colon cell with an elevated expression of integrin αvβ3.

The uptake of G-SP@MCaP by HCT116/L cells and its mechanism were investigated by laser scanning confocal microscopy (LSCM) and flow cytometry. To simulate the microenvironment of orthotopic drug-resistant colon cancer, macrophages (RAW264.7), normal colon epithelial cells (NCM460), and drug-resistant colon cancer cells (HCT116/L) were co-cultured. Then, these cells were co-incubated with G-SP@MCaP. Compared with SP@MCaP, the accumulation of G-SP@MCaP in RAW264.7 and NCM460 cells was significantly diminished, while the accumulation in HCT116/L cells increased in a time-dependent manner (Fig. 2D-G and Fig.S5). Besides, immunofluorescence staining revealed a high overlap between Cy5-labeled G-SP@MCaP and integrin αvβ3 in HCT116/L cells (Fig. 2H). Furthermore, the uptake experiment indicated that G-SP@MCaP was ingested by HCT116/L cells primarily through caveolin pathway and clathrin pathway. After pretreatment with Gal-3 or c(RGDfk) cyclic peptide (blocker of integrin αvβ3), the uptake of G-SP@MCaP by HCT116/L cells was markedly inhibited (Fig. 2I-J and Fig.S6). These findings suggested that the uptake of G-SP@MCaP by HCT116/L cells was mediated through integrin αvβ3. In contrast, RAW264.7 and NCM460 cells exhibited a reduced uptake of G-SP@MCaP, which resulted from low expression of integrin αvβ3 in these cells.

After entering the cell, siRNA is susceptible to degradation by enzymes in lysosomes. Therefore, efficient escape of siRNA from lysosomes is essential for the silencing effect of siRNA delivery system [40]. G-SP@MCaP could gradually dissolve in the acidic environment of lysosomes, releasing a large amount of Ca^2+^ and PO_4_^3−^. This led to an increased permeability of lysosome membrane and allowed for the escape of siGal-3 from lysosomes. As shown in Fig. 2K, siGal-3 mainly accumulated in lysosomes after incubating G-SP@MCaP with HCT116/L cells for 2 h. However, there was a significant reduction in the overlap between siGal-3 and lysosomes after incubation for 4 h. These results confirmed that G-SP@MCaP facilitated the escape of siGal-3 from lysosomes. As a result, G-SP@MCaP could effectively inhibit the expression of Gal-3 in HCT116/L cells (Fig. 2L).

**Fig. 3:**
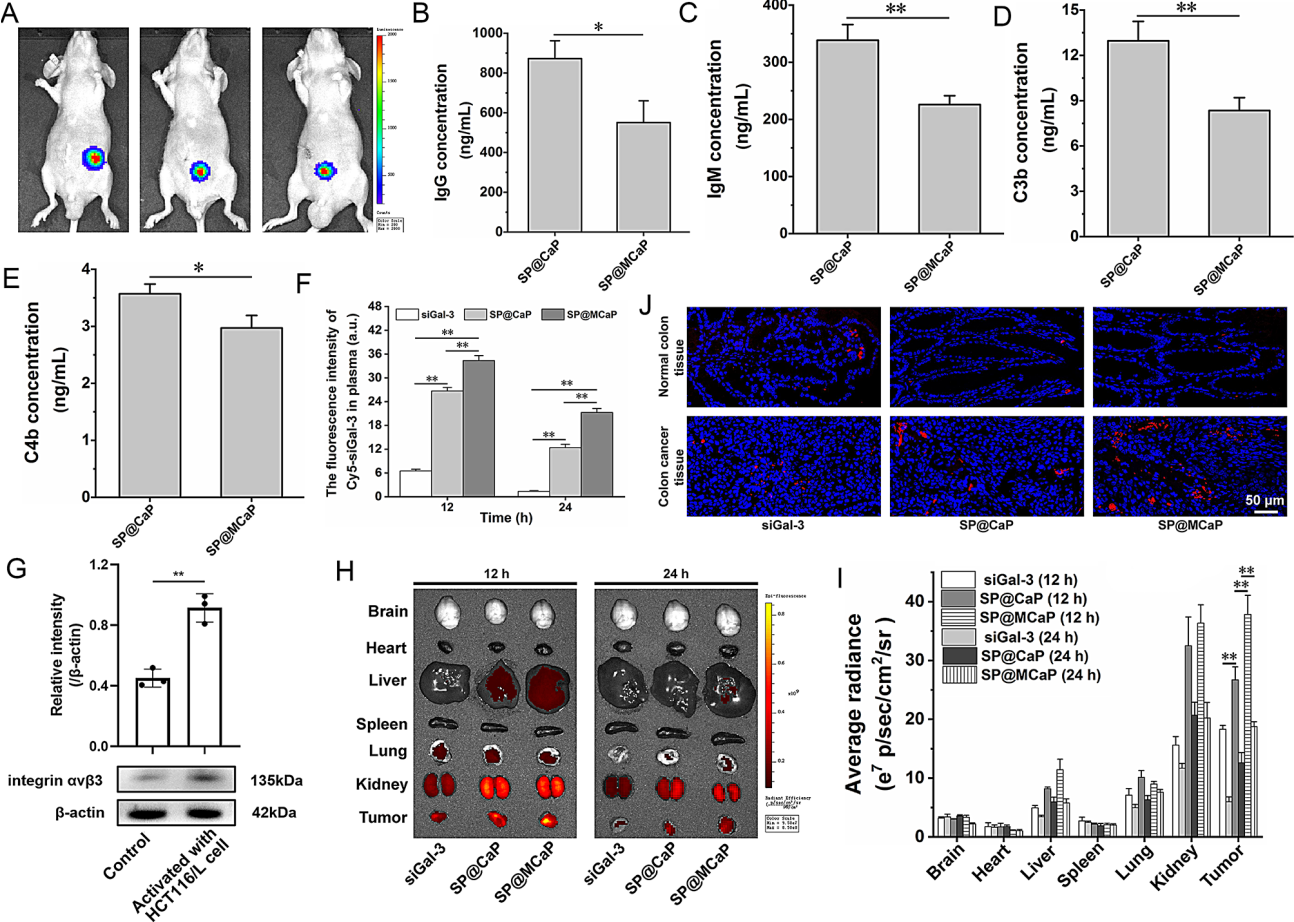
SP@MCaP distribution in nude mice with orthotopic drug-resistant colon cancer. (**A**) Fluorescent images of orthotopic drug-resistant colon cancer in nude mice. (**B-E**) The adsorption of immune-related proteins and complement activated fragments by SP@MCaP. (**F**) Dynamic change of fluorescence intensity in serum of nude mice with orthotopic drug-resistant colon cancer. (**G**) Expression of integrin αvβ3 in normal HUVEC cells and HCT116/L activated HUVEC cells. (**H-I**) Distribution of SP@MCaP in organs and cancer tissues of nude mice with orthotopic drug-resistant colon cancer. (**J**) Typical LSCM picture of distribution of SP@MCaP in orthotopic drug-resistant colon cancer tissues and normal colon tissues from nude mice with orthotopic drug-resistant colon cancer. *n* = 3, mean ± SD, ^*^P < 0.05, ^**^P < 0.01

Following intraperitoneal injection of D-luciferin potassium into nude mice with orthotopic drug-resistant colon cancer, D-luciferin potassium produces biofluorescence under the action of luciferase in HCT116/L-luc cells. The fluorescent signals was detected in the lower abdomen of nude mice by in vivo imager (Fig. 3A). This indicated that nude mice model with orthotopic drug-resistant colon cancer was successful established. When nanoparticles enter the blood circulation, they are prone to rapid clearance by the mononuclear phagocyte system, leading to a weakened therapeutic effect [41]. Studies have demonstrated that the formation of protein corona on the surface of nanoparticles can significantly decrease the adsorption of IgG and complement activated fragments (C3b, C4b), thereby increasing their stability in blood circulation [42]. When incubation with serum from nude mice with orthotopic drug-resistant colon cancer, SP@MCaP showed less adsorption capacity on immune-related proteins (IgG and IgM) and complement activated fragments (C3b and C4b) than that of SP@CaP (Fig. 3B-E). This phenomenon was attributed to the active capture of Gal-3 by SP@MCaP in serum and formation of a protein corona on its surface. Therefore, after intravenous injection via tail vein, the fluorescence intensity of SP@MCaP in serum was significantly higher than that of free siGal-3 and SP@CaP at 12 and 24 h (Fig. 3F).

The expression of integrin αvβ3 in normal HUVEC cells and HCT116/L cells culture medium-treated HUVEC cells was investigated by western blot. Notably, the experimental findings indicated that there was a higher amount of integrin αvβ3 in HCT116/L cells culture medium-treated HUVEC cells than that in normal HUVEC cells (Fig. 3G). This suggested that cancer cells stimulated the expression of integrin αvβ3 in vascular endothelial cell. Therefore, as compared to SP@CaP, SP@MCaP selectively accumulated in drug-resistant colon cancer tissue in which integrin αvβ3 was highly expressed (Fig. 3H-J). These findings demonstrated that SP@MCaP could actively capture Gal-3 in serum and form a Gal-3 protein corona on its surface. This helped SP@MCaP to evade recognition and clearance by the mononuclear phagocyte system, prolonging the circulation time of SP@MCaP in bloodstream. Then, the Gal-3 protein corona on SP@MCaP actively recognized integrin αvβ3 on vascular endothelial cell in cancer tissue and drug-resistant colon cancer cells. Consequently, SP@MCaP accumulated at orthotopic drug-resistant colon cancer tissue.

### G-SP@MCaP significantly inhibited the proliferation of HCT116/L cells by activating the mitochondrial apoptotic pathway

**Fig. 4:**
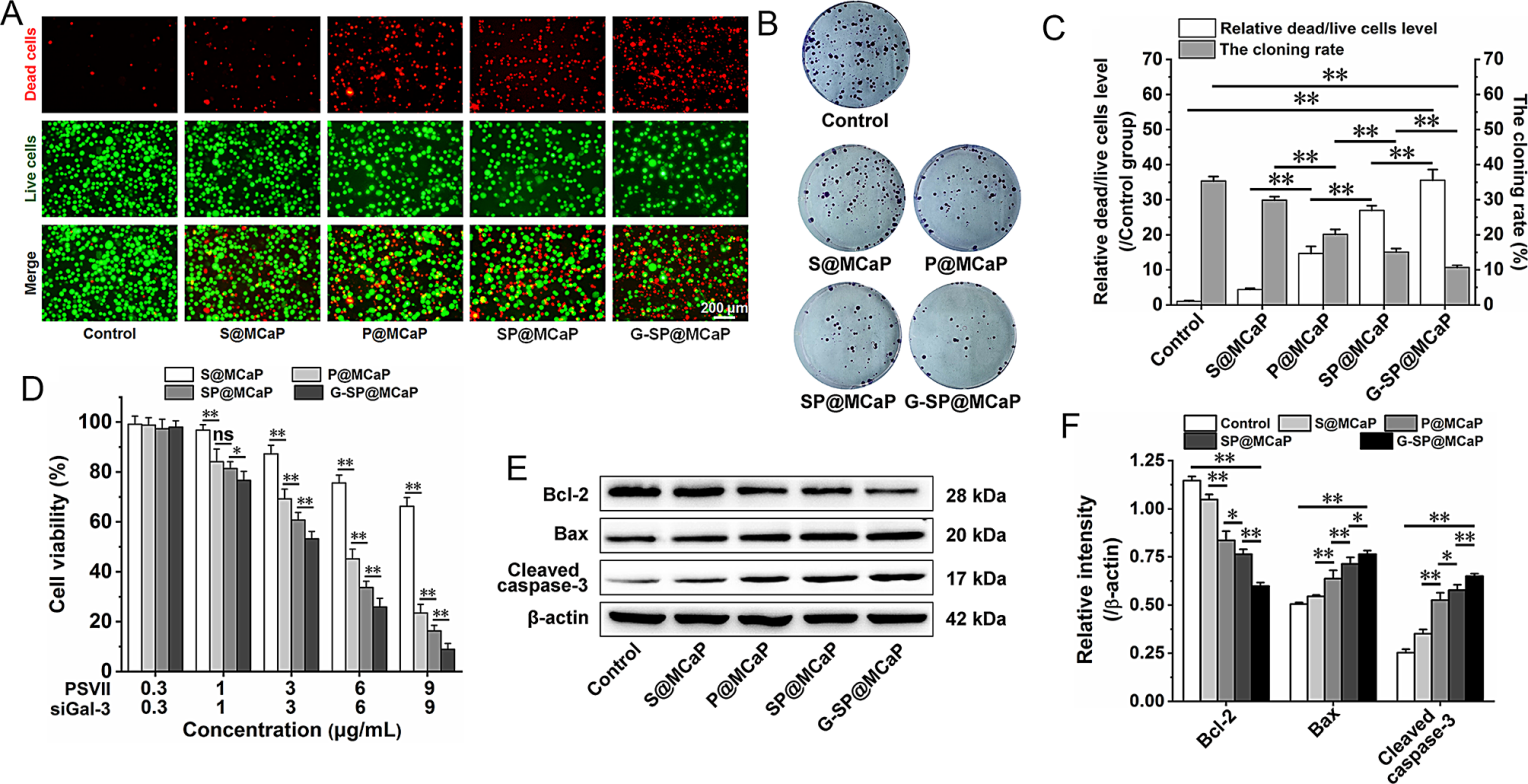
Effect of G-SP@MCaP on the proliferation of HCT116/L cells. (**A-C**) The dead/living cell staining and cloning formation of HCT116/L cells. (**D**) MTT results of G-SP@MCaP on HCT116/L cells. (**E-F**) The expression of apoptosis-related protein. *n* = 3, mean ± SD, ^*^P < 0.05, ^**^P < 0.01, ns: no significant difference

The results of dead/living cell staining, cell cloning formation and MTT assay showed that S@MCaP, P@MCaP, SP@MCaP and G-SP@MCaP significantly suppressed the proliferation of HCT116/L cells (Fig. 4A-D). Notably, P@MCaP exhibited an enhanced inhibitory effect on the proliferation of HCT116/L cells as compared to S@MCaP. SP@MCaP exhibited higher inhibitory effect on the proliferation of HCT116/L cells as compared with P@MCaP. Among all groups, G-SP@MCaP displayed the strongest inhibitory activity against HCT116/L cell proliferation. Moreover, western blot results revealed that G-SP@MCaP significantly increased the expression of Bax and Cleaved caspase-3 while decreased the expression of Bcl-2 as compared with S@MCaP, P@MCaP and SP@MCaP (Fig. 4E-F). These findings suggested that siGal-3 and PSVII exerted a synergistic effect on inhibiting the proliferation of HCT116/L cells. Meanwhile, G-SP@MCaP inhibited the proliferation by activating the mitochondrial apoptotic pathway in HCT116/L cells [43].

### G-SP@MCaP significantly inhibited the migration and invasion through regulating the adhesion of HCT116/L cells

**Fig. 5:**
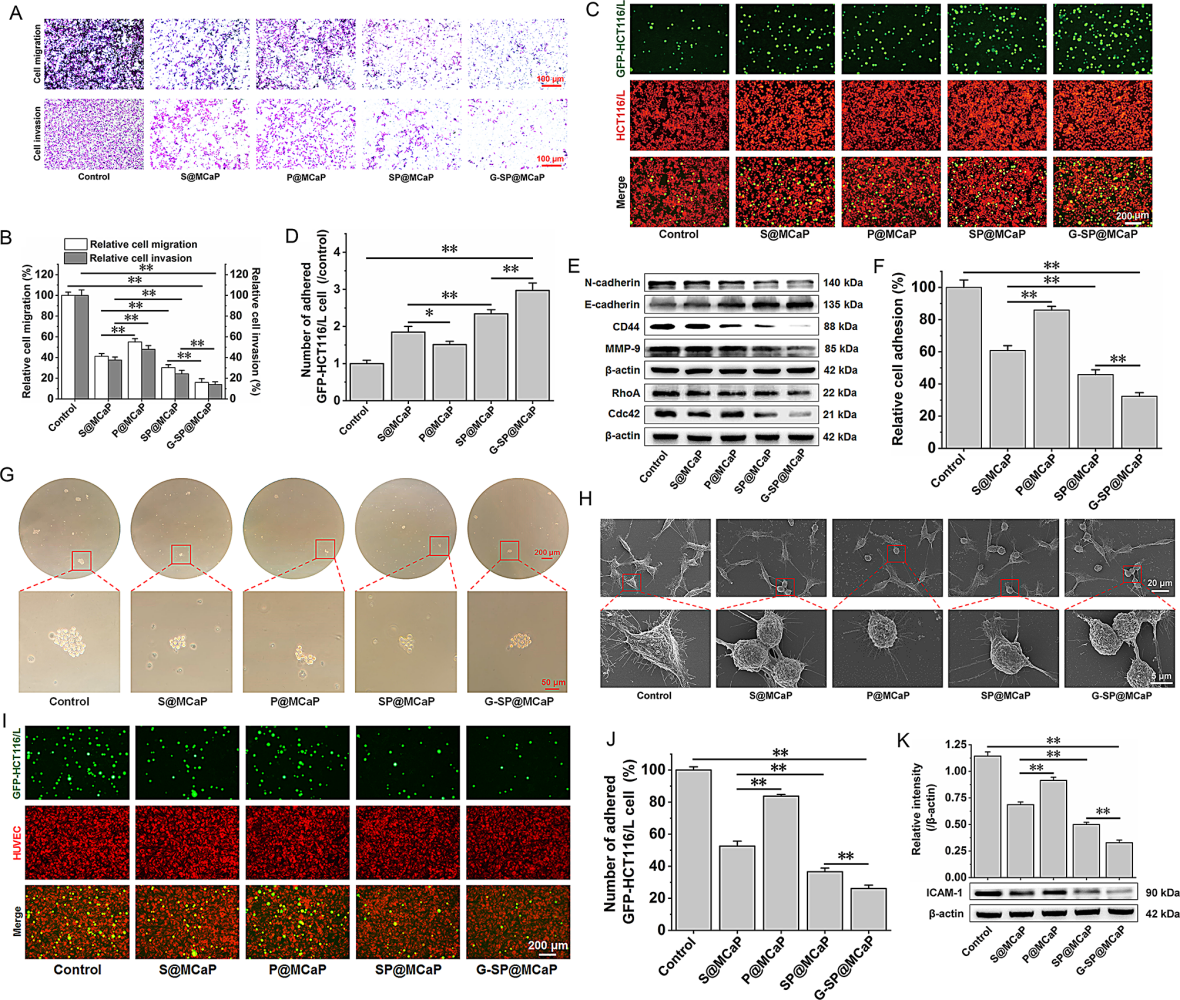
Effects of G-SP@MCaP on adhesion of HCT116/L cells. (**A-B**) The migration and invasion of HCT116/L cells. (**C-D**) Intercellular adhesion between HCT116/L cells. (**E**) The expression of invasion-related protein and motion-related protein in HCT116/L cells. (**F**) Effect of G-SP@MCaP on adhesion between HCT116/L cells and matrix. (**G**) Typical picture of homotypic aggregation of HCT116/L cells. (**H**) Typical picture of morphology and pseudopodia formation of HCT116/L cells. (**I**) Typical picture of adhesion between HCT116/L cells and HUVEC cells. (**J**) Statistical results of adhesion between HCT116/L cells and HUVEC cells. (**K**) ICAM-1 expression in HUVEC cells. *n* = 3, mean ± SD, ^*^P < 0.05, ^**^P < 0.01

The Gal-3 outside cancer cells binds to the integrins that are highly expressed on the surface of cancer cells, leading to close adhesion of cancer cells to laminin and fibronectin in the surrounding matrix. This supplies a fulcrum for the movement of detached single cancer cells and promote the invasion of cancer cells. The experimental results demonstrated that SP@MCaP significantly suppressed the invasion and migration of HCT116/L cells in comparison to S@MCaP and P@MCaP. Compared with SP@MCaP, G-SP@MCaP exhibited stronger inhibitory activity against HCT116/L cell invasion and migration (Fig. 5A-B).

The uncontrolled adhesion and proliferation of cancer cells are key factors leading to cancer cell invasion and metastasis [44]. Inhibiting the expression of Gal-3 can reconstruct the adhesion of cancer cells and suppress their invasion and metastasis. Due to the replacement of E-cadherin with N-cadherin in cancer cells, more flexible connections are formed between cancer cells [45]. This results in weakened adhesion and easier separation among cancer cells. Additionally, cancer cells shed from cancer tissues can bind to Gal-3 outside the cell through adhesion molecules such as integrin αvβ3 on its surface. This allows them to tightly adhere to fibulin and laminin in the surrounding matrix and move around [46, 47]. At the same time, MMP-9 secreted by cancer cells can continuously degrade the extracellular matrix, and then damage the basement membrane. Subsequently, the invasion and metastasis of cancer cells is occurred [48]. The adhesion experiment showed that the number of GFP-HCT116/L cells attached to HCT116/L cells increased significantly when pretreatment with SP@MCaP. Furthermore, compared with SP@MCaP, G-SP@MCaP further increased the number of attached GFP-HCT116/L cells (Fig. 5C-D). Western blot results revealed that G-SP@MCaP significantly enhanced the expression of E-cadherin, while decreased the expression of N-cadherin and MMP-9 in HCT116/L cells (Fig. 5E and Fig.S7A). These findings suggested that G-SP@MCaP enhanced the adhesion between HCT116/L cells by increasing the expression of E-cadherin and decreasing the expression of N-cadherin and MMP-9. This resulted in the inhibition of migration and invasion of HCT116/L cells.

The impact of G-SP@MCaP on the adhesion between HCT116/L cells and matrix was also investigated. The number of HCT116/L cells adhered to matrix decreased significantly in all drug treated groups. In comparison to S@MCaP and SP@MCaP, G-SP@MCaP notably reduced the adhesion ability between HCT116/L cells and matrix (Fig. 5F). These results indicated that G-SP@MCaP effectively inhibited the invasion and metastasis by decreasing the adhesion of HCT116/L cells to matrix.

Free Gal-3 in the blood facilitates the polarization of mucin 1 (MUC1) on the surface of circulating cancer cells, thereby exposing cell adhesion molecules. This enhanced the homotypic aggregation among circulating cancer cells to form cancer thrombus [49, 50]. The cancer thrombus not only withstands the high shear stress of the blood flow, but also provides protection against attacks from the immune system [51]. The influence of G-SP@MCaP on the formation of HCT116/L cell thrombus was investigated by examining the formation of cancer cell aggregates in vitro. As shown in Fig. 5G, the aggregates in the control group were large and numerous. Nevertheless, the aggregates in the drug treated groups were small and limited. In comparison to other formulation, G-SP@MCaP showed no significant reduction in the number and size of aggregates. This attributed to the fact that G-SP@MCaP already captured Gal-3 on the surface of itself, thereby forfeiting its capacity to capture free Gal-3 in the culture medium. These results suggested that nanoparticles containing MCP had the ability to capture free Gal-3 in the culture medium, leading to a reduction in the level of Gal-3 in the culture medium. Then, the homotypic aggregation of HCT116/L cells was inhibited.

Gal-3 can enhance the formation of pseudopodia in human colon cancer cells, thereby facilitating invasion and metastasis [52, 53]. Therefore, the effects of G-SP@MCaP on cell morphology and pseudopodia of HCT116/L cells were observed by SEM. The results revealed that untreated HCT116/L cells exhibited tight connections, small gaps, flat and polygonal shapes, and dense pseudopodia. However, after treatment with G-SP@MCaP, HCT116/L cells became more spherical in shape. At the same time, HCT116/L cells showed looser intercellular connection, enlarged space, and fewer sparse pseudopodia (Fig. 5H). In addition, western blot results showed that G-SP@MCaP significantly diminished the expression of motion-related protein (RhoA and Cdc42) in HCT116/L cells (Fig. 5E and Fig.S7B). These results suggested that G-SP@MCaP inhibited the invasion and metastasis by decreasing pseudopodia formation and movement of HCT116/L cells.

The adhesion of cancer cells to vascular endothelial cells in metastatic target organs is a crucial step in the process of cancer cell metastasis [54]. Circulating cancer cells can activate vascular endothelial cells to secrete intercellular adhesion molecule-1 (ICAM-1) and vascular cell adhesion factor-1 (VCAM-1), thus enhancing the adhesion between circulating cancer cells and vascular endothelial cells. This promoted cancer cell metastasis [55]. Gal-3 plays a crucial role in promoting the adhesion of cancer cells with vascular endothelial cells in metastatic target organs by regulating the adhesion of cancer cells. Adhesion experiment showed when HCT116/L cells and HUVEC cells were co-cultured, they adhered together in large quantities. However, after pretreatment with G-SP@MCaP, there was a significant reduction in the number of HCT116/L cells attached to HUVEC cells (Fig. 5I-J). Furthermore, it was found that HUVEC cell highly expressed ICAM-1 when co-cultured with HCT116/L cells. Interestingly, pretreatment of HCT116/L cells with G-SP@MCaP decreased the expression of ICAM-1 in HUVEC cells when co-cultured with HCT116/L cells (Fig. 5K). These results indicated that G-SP@MCaP reduced the activation of HUVEC cells induced by HCT116/L cells through decreasing the level of Gal-3 inside and outside of HCT116/L cells. This ultimately weakened the adhesion between HCT116/L cells and HUVEC cells. The above results suggested that G-SP@MCaP diminished the adhesion ability of HCT116/L cells to the vascular endothelium of the target organ as well as the metastasis ability of HCT116/L cells.

### SP@MCaP significantly inhibited the growth of orthotopic drug-resistant colon cancer in nude mice by inducing apoptosis of cancer cells and inhibiting angiogenesis

**Fig. 6:**
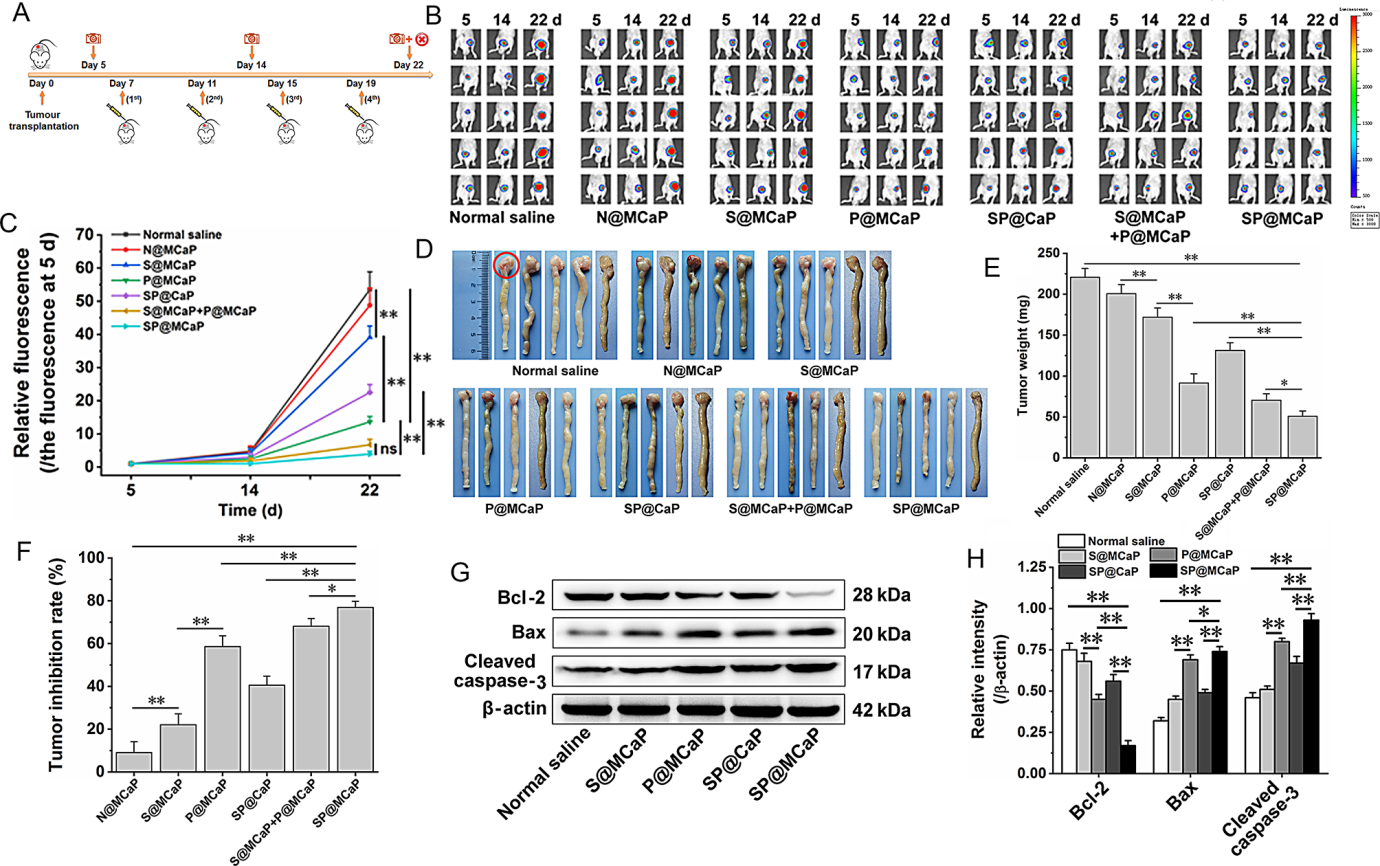
Inhibitory effect of SP@MCaP on orthotopic drug-resistant colon cancer in nude mice. (**A**) Schematic diagram of treatment regimen for nude mice with orthotopic drug-resistant colon cancer. (**B-C**) The growth of orthotopic drug-resistant colon cancer tissues in nude mice during treatment, *n* = 5. (**D**) The typical pictures of colon cancer tissue (red circle indicates the location of drug-resistant colon cancer tissue), *n* = 5. (**E**) The weight of colon cancer tissue, *n* = 3. (**F**) Inhibition rate for orthotopic drug-resistant colon cancer, *n* = 3. (**G-H**) The expression of apoptosis-related protein in drug-resistant colon cancer tissue, *n* = 3. Mean ± SD, ^*^P < 0.05, ^**^P < 0.01

To explore the anti-cancer activity of SP@MCaP in vivo, we established a nude mouse model of orthotopic drug-resistant colon cancer. The cancer-bearing mice were treated as outlined in Fig. 6A. The results revealed that S@MCaP exhibited low activity against the growth of orthotopic drug-resistant colon cancer (Fig. 6B-F). P@MCaP exhibited a considerable inhibitory effect on the growth of orthotopic drug-resistant colon cancer, while SP@MCaP displayed the strongest inhibitory effect on the growth of orthotopic drug-resistant colon cancer. It was worth noting that SP@CaP did not contain MCP, so it could not capture Gal-3 in blood, which subsequently resulted in less distribution in the tissues of orthotopic drug-resistant colon cancer. Consequently, compared with SP@MCaP, SP@CaP exhibited a weaker inhibitory effect on the growth of orthotopic drug-resistant colon cancer. In the normal saline group, drug-resistant colon cancer grew rapidly, leading to a significant weight loss in nude mice. SP@MCaP ameliorated the weight loss of cancer-bearing nude mice (Fig.S8).

**Fig. 7:**
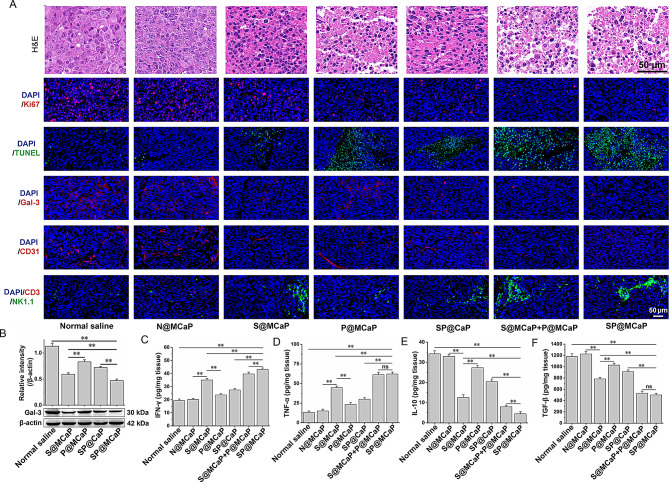
Effects of SP@MCaP on apoptosis and immune microenvironment of orthotopic drug-resistant colon cancer tissues. (**A**) Typical picture of *H&E* and immunofluorescence staining of Ki67, Gal-3, CD31, CD3/NK1.1 and TUNEL^+^ cells in orthotopic drug-resistant colon cancer tissues. (**B**) The expression of Gal-3 in orthotopic drug-resistant colon cancer tissues. (**C-F**) The contents of IFN-γ, TNF-α, IL-10 and TGF-β in orthotopic drug-resistant colon cancer tissues. *n* = 3, mean ± SD, ^**^P < 0.01, ns: no significant difference

Western blot results revealed that SP@MCaP significantly increased the expression of Bax and Cleaved caspase-3, while decreased the expression of Bcl-2 in orthotopic drug-resistant colon cancer tissues (Fig. 6G-H). *H&E* staining indicated that there were obvious atypic cells and pathological mitotic images with a high number of atypic nuclei in the normal saline group. Conversely, the number of cancer cells in cancer tissues was notably reduced, accompanied by evident nuclear shrinkage and nuclear reduction in the SP@MCaP group. Ki67 staining showed that a high level of Ki67 was expressed in the normal saline group, indicating rapid proliferation of orthotopic drug-resistant colon cancer cells. Moreover, as compared with S@MCaP, P@MCaP and SP@CaP, SP@MCaP significantly decreased Ki67 expression, suggesting proliferation of orthotopic drug-resistant colon cancer cells was suppressed by SP@MCaP. Simultaneously, TUNEL staining results also revealed that the number of apoptotic cells (TUNEL^+^ cells) was the highest in SP@MCaP group (Fig. 7A). These results suggested that SP@MCaP impeded the growth of orthotopic drug-resistant colon cancer by promoting the apoptosis of orthotopic drug-resistant colon cancer cells.

The results of western blot and immunofluorescence staining revealed that S@MCaP and SP@MCaP significantly diminished the level of Gal-3 in orthotopic drug-resistant colon cancer tissues (Fig. 7A-B). This was attributed to the silencing of Gal-3 expression caused by SP@MCaP in orthotopic drug-resistant colon cancer tissue. Additionally, SP@MCaP markedly increased the expression of E-cadherin, while decreased the expression of CD44, N-cadherin, MMP-9, RhoA, and Cdc42 in orthotopic drug-resistant colon cancer tissues (Fig.S9). These results demonstrated that SP@MCaP reduced the degradation of extracellular matrix and enhanced the adhesion between cancer cells in orthotopic drug-resistant colon cancer tissues. This inhibited the shedding of cancer cells and the motility of cancer cells. Consequently, SP@MCaP exhibited great potential in impeding the metastasis of orthotopic drug-resistant colon cancer.

It is known that Gal-3 activates vascular endothelial growth factor (VEGF) signaling pathway to promote angiogenesis by binding with integrin αvβ3 on vascular endothelial cells [56]. Blood vessels play a crucial role in providing continuous supply of nutrients for the growth and spread of cancer cells [57], thereby facilitating their proliferation and metastasis. Studies have shown that Gal-3 can stimulate angiogenesis mediated by VEGF and basic fibroblast growth factor [58], which in turn promotes the growth of cancer cells. The tubule formation of HUVEC cells showed that a large number of intact tubules were formed in the control group, while the drug treated group showed a significant reduction in tubule formation (Fig.S10). Furthermore, the number of tubules in S@MCaP group was notably lower than that in P@MCaP group, and the number of tubules in G-SP@MCaP group was the least. These results suggested that G-SP@MCaP could effectively suppress the tubule formation of HUVEC cells by downregulating Gal-3 expression and ultimately inhibit angiogenesis in cancer tissues. In vivo, a large amount of CD31^+^ cells were observed in orthotopic drug-resistant colon cancer tissues in normal saline group (Fig. 7A), indicating abundant vascular growth. Compared with P@MCaP, both SP@MCaP and S@MCaP significantly inhibited angiogenesis in orthotopic drug-resistant colon cancer tissues. In summary, the above data demonstrated that SP@MCaP and S@MCaP inhibited the growth of orthotopic drug-resistant colon cancer by reducing blood vessel formation.

Studies have demonstrated that Gal-3 binds to glycoprotein receptors on the surface of T cells, leading to T cell aggregation and the decrease of T cell activity [59]. In addition, high expression of Gal-3 in cancer tissues can shield NK cell binding sites on the surface of cancer cells, thereby reducing the killing effect of NK cells on cancer cells [60]. The experimental results revealed a significant increase in the recruitment of NK cells within orthotopic drug-resistant colon cancer tissues following treatment with SP@MCaP (Fig. 7A). Concurrently, there was a marked elevation in the levels of TNF-α and IFN-γ, while there was a notable reduction in the levels of TGF-β and IL-10 in orthotopic drug-resistant colon cancer tissues (Fig. 7C-F). These results indicated that SP@MCaP promoted NK cells recruitment by downregulating the level of Gal-3 in drug-resistant colon cancer tissues. SP@MCaP significantly ameliorated the immunosuppressive microenvironment in orthotopic drug-resistant colon cancer tissues, subsequently inhibited the growth of orthotopic drug-resistant colon cancer.

The above results demonstrated that SP@MCaP promoted the apoptosis of drug-resistant colon cancer cells and reduced blood vessel formation. Meanwhile, the immunosuppressive microenvironment was reversed in orthotopic drug-resistant colon cancer tissues. Finally, the growth of orthotopic drug-resistant colon cancer was inhibited.

### SP@MCaP significantly inhibited the liver metastases of colon cancer by regulating the adhesion of HCT116/L cells and attenuating the activation of liver vascular endothelial cells

**Fig. 8:**
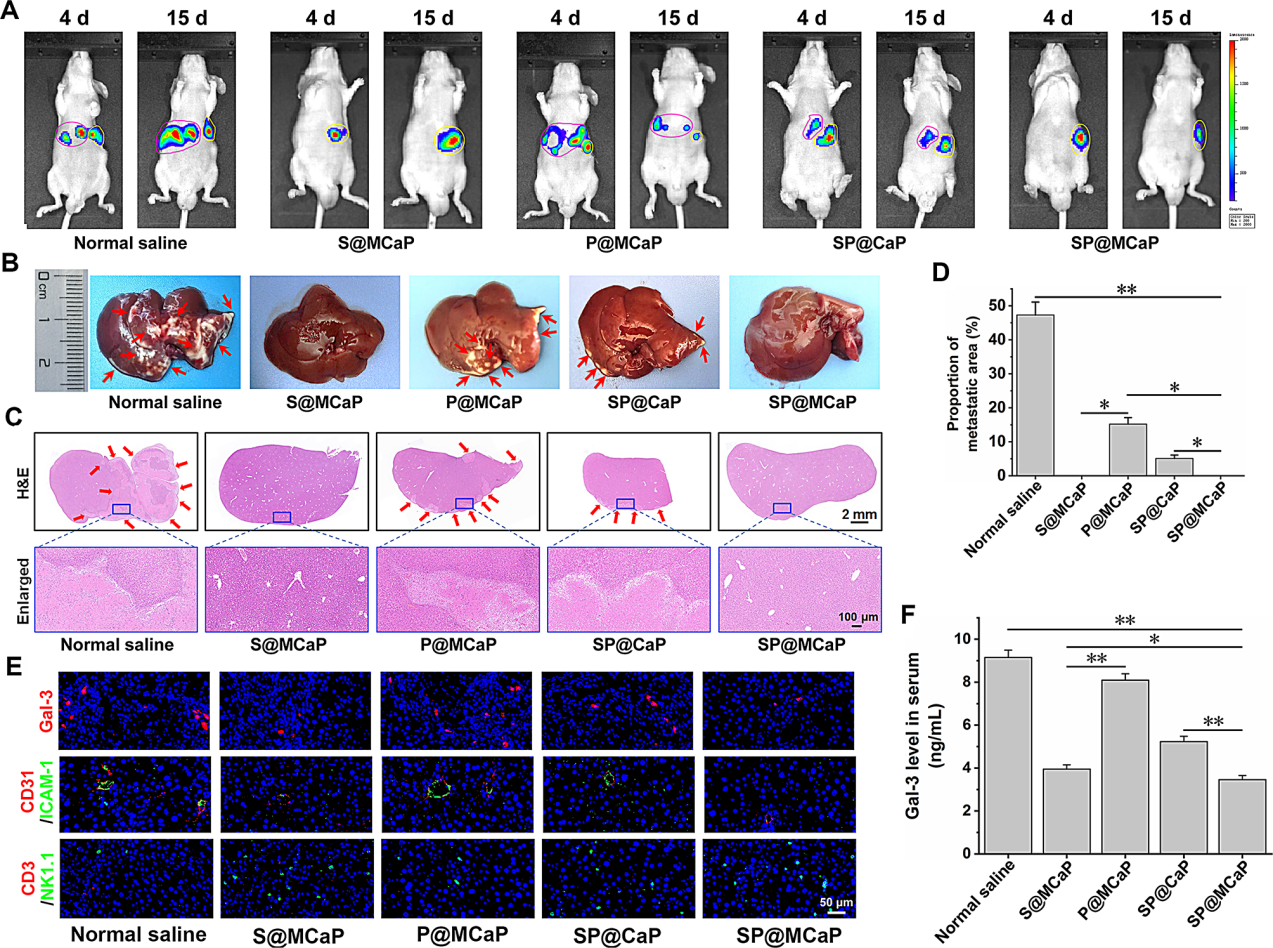
Effect of SP@MCaP on liver metastases of drug-resistant colon cancer cells. (**A**) Typical in vivo image of nude mice with liver metastasis of drug-resistant colon cancer (yellow circle indicates splenic tumors, and pink circle indicates hepatic tumors). (**B**) Typical pictures of cancer nodule in liver of nude mice (The red arrow represents hepatic metastasis). (**C**) Typical *H&E* staining pictures of liver tissue of nude mice (The red arrow represents the lesion of hepatic metastases). (**D**) Statistical results of the proportion of liver metastases. (**E**) Typical picture of immunofluorescence staining of Gal-3, CD31/ICAM-1 and NK cells (CD3^−^/NK1.1^+^) in liver tissue of nude mice. (**F**) The content of Gal-3 in serum of cancer-bearing nude mice. *n* = 3, mean ± SD, ^*^P < 0.05, ^**^P < 0.01

Following the injection of HCT116/L cells into the spleen, there was a notable proliferation of cancer cells, leading to their migration to the liver via the portal vein. The in vivo imaging revealed that compared with normal saline group, there was distinct fluorescence signal in liver in P@MCaP and SP@CaP treated groups, indicating P@MCaP and SP@CaP did not impede the liver metastasis of drug-resistant colon cancer cells. However, no significant fluorescence signal was observed in liver in S@MCaP and SP@MCaP treated groups (Fig. 8A), suggesting that these formulations effectively blocked liver metastases of drug-resistant colon cancer cells. Upon isolation of livers from nude mice, there was no obvious cancer nodule in the livers in S@MCaP and SP@MCaP treated groups. However, noticeable cancer nodules were observed in the livers in normal saline, P@MCaP and SP@CaP treated groups (Fig. 8B). In addition, the *H&E* staining results also demonstrated the excellent inhibitory effect of SP@MCaP on liver metastasis (Fig. 8C-D).

Clinical studies have shown that the level of Gal-3 in cancer tissue and serum of patients with colon cancer is significantly elevated. The higher the level of serum Gal-3, the more likely colon cancer metastasis [19]. Immunofluorescence staining and ELISA results revealed that SP@MCaP significantly reduced the level of Gal-3 in liver tissue and serum, resulted in an increased recruitment of NK cells in liver tissue (Fig. 8E-F). This indicated that SP@MCaP reversed the immunosuppressive microenvironment of liver by decreasing Gal-3 level. In addition, SP@MCaP diminished the expression of ICAM-1 in the blood vessels of liver tissues (Fig. 8E), which decreased the adhesion of circulating drug-resistant colon cancer cells with blood vessels in liver tissues. Finally, the liver metastasis of drug-resistant colon cancer cells was blocked.

The above results demonstrated when SP@MCaP entered the bloodstream, it automatically captured Gal-3 in the bloodstream and obtained a targeting ligand. Subsequently, it adhered to circulating drug-resistant colon cancer cells through integrin αvβ3 and then killed them. Simultaneously, SP@MCaP diminished the adhesion between HCT116/L cells in blood circulation and the formation of cancer thrombi. This promoted the anoikis of circulating drug-resistant colon cancer cells. Moreover, SP@MCaP decreased serum Gal-3 level by directly capturing Gal-3 in the blood. This attenuated the activation of vascular endothelial cells in liver, thereby inhibiting the adhesion of circulating drug-resistant colon cancer cells to vascular endothelial cells. Ultimately, SP@MCaP inhibited the liver metastases of drug-resistant colon cancer cells.

## Conclusions

In summary, MCP serves as a natural ligand of Gal-3, thus SP@MCaP exhibited high affinity with Gal-3. By automatically capturing Gal-3 in the blood circulation, SP@MCaP could diminish Gal-3 level in serum and form protein corona on the surface of nanoparticles (G-SP@MCaP). G-SP@MCaP kept stability in neutral environment and had distinct pH-responsive drug release characteristics. The diminished Gal-3 level in serum by G-SP@MCaP weakened the activation of vascular endothelial cells. This subsequently inhibited the adhesion of drug-resistant colon cancer cells in the blood circulation with vascular endothelial cells in the metastatic target organs. The protein corona on the surface of nanoparticles decreased the adsorption of immune-related proteins (IgG and IgM) and complement activated fragments (C3b and C4b), thereby increasing their stability in blood circulation. Subsequently, G-SP@MCaP selectively accumulated in drug-resistant colon cancer cells with elevated integrin αvβ3 expression and simultaneously released siGal-3 and PSVII. PSVII directly promoted the apoptosis of orthotopic drug-resistant colon cancer cells. Meanwhile, siGal-3 decreased the expression of Gal-3, subsequently attenuated the inhibitory effect of Gal-3 on cell apoptosis, and then strengthened the effect of PSVII on inducing the apoptosis of drug-resistant colon cancer cells. Besides, the adhesion between drug-resistant colon cancer cells was enhanced, and the adhesion between drug-resistant colon cancer and vascular endothelial cells was deceased by siGal-3. Furthermore, immunosuppressive microenvironment in orthotopic drug-resistant colon cancer and liver was reversed by decreasing Gal-3 level. Ultimately, the simultaneous delivery of siGal-3 and PSVII synergistically suppressed the proliferation and metastasis of drug-resistant colon cancer by remodeling cell adhesion and immune microenvironment, as well as promoting apoptosis of drug-resistant colon cancer cells. This strategy holds great promise for the targeted and synergistic therapy of drug-resistant colon cancer.

## Electronic supplementary material

Below is the link to the electronic supplementary material.

Supplementary Material 1

## Data Availability

No datasets were generated or analysed during the current study.

## Abbreviations

ALN: Alendronate
ALT: Alanine aminotransferase
AST: Aspartate aminotransferase
Bax: Bcl-2-associated x protein
BCA: Bicinchoninic acid
Bcl-2: B-cell lymphoma-2
BUN: Blood urea nitrogen
CD44: Cluster of differentiation 44
Cdc42: Cell division control protein 42
Cleaved caspase-3: Cleaved cysteine protease 3
CREA: Creatinine
DAPI: 4’,6-Diamidino-2-phenylindole
ELISA: Enzyme-linked immunosorbent assay
Gal-3: Galectin-3
IFN-γ: Interferon-γ
IgG: Immunoglobulin G
IgM: Immunoglobulin M
IL-10: Interleukin-10
LAMP1: Lysosome associated membrane protein 1
LSCM: Laser scanning confocal microscopy
MCP: Modified citrus pectin
MTT: 3-(4,5-Dimethylthiazol-2-yl)-2,5-diphenyltetrazolium bromide
NK cells: Natural killer cell
PBS: Phosphate buffered saline
PSVII: Paris sponin VII
RhoA: RAS homolog gene family member A
SEM: Scanning electron microscope
TEM: Transmission electron microscope
TNF-α: Tumor necrosis factor-α
UPLC: Ultra-high performance liquid chromatography
