# Supplementary material for: Automatic target-seeking nanoparticle inhibiting orthotopic drug-resistant colon cancer and liver metastases via regulating cancer cell adhesion and proliferation
Source: J Nanobiotechnology. 2025 Jun 6;23:423. doi: 10.1186/s12951-025-03422-x (PMC12142993; doi:10.1186/s12951-025-03422-x)
Supplement: Supplementary file 1 — Supplementary Material 1 [file 12951_2025_3422_MOESM1_ESM.docx]

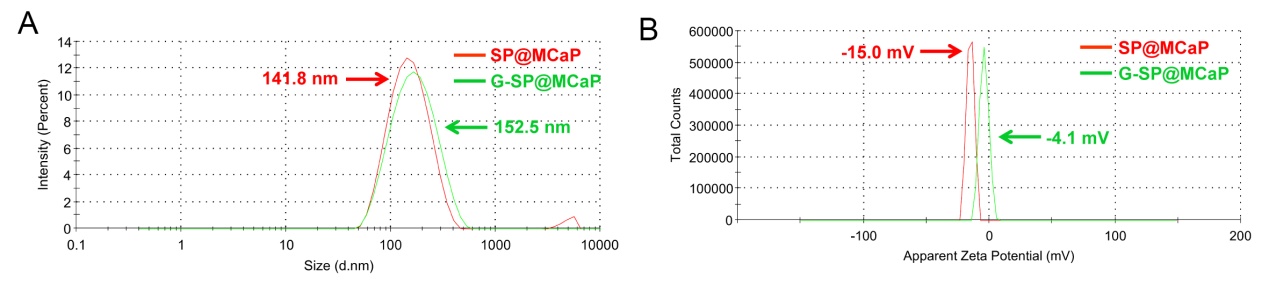


**Fig. S1.** Particle size (A) and Zeta potential (B) of SP@MCaP and G-SP@MCaP.


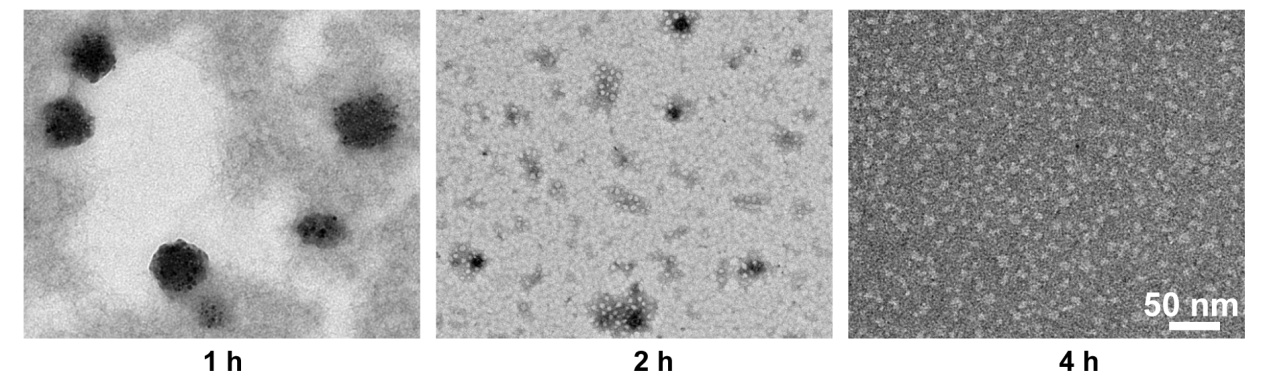


**Fig. S2.** The corrosion of G-SP@MCaP under pH 5.0 medium.


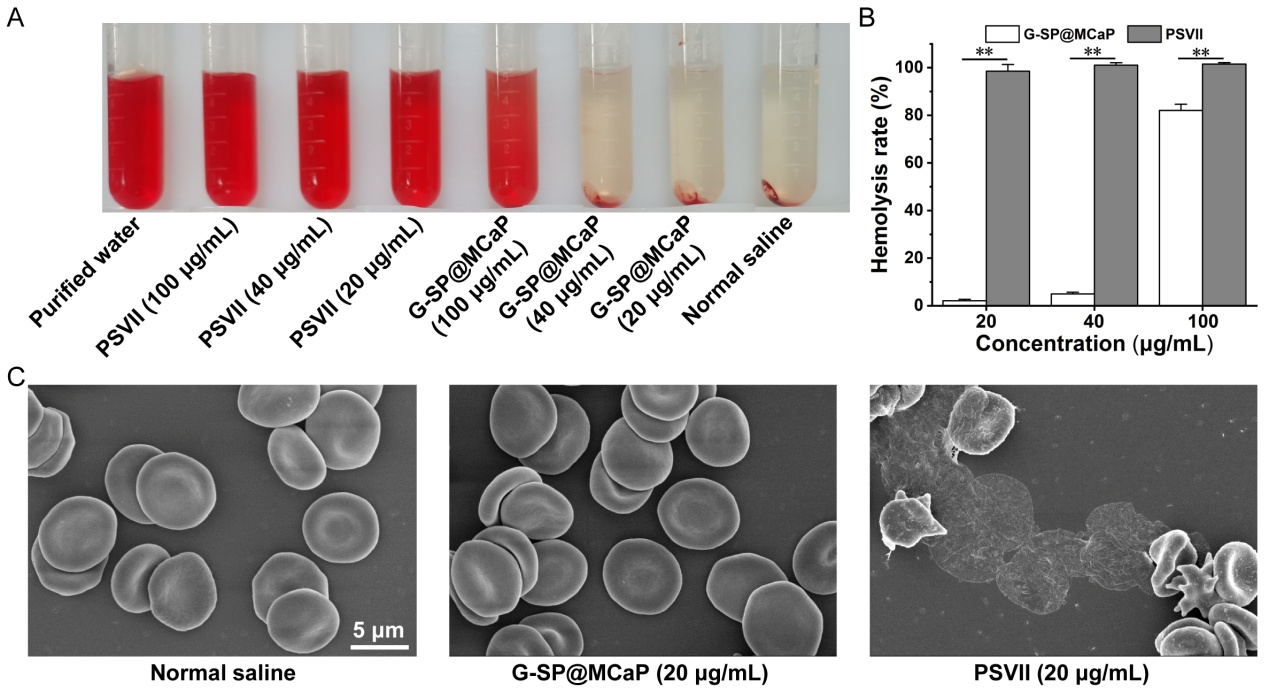


**Fig. S3.** The hemolysis effect of G-SP@MCaP. (A) Typical pictures of hemolysis. (B) Statistical results of hemolysis rate. (C) Typical picture of erythrocyte membrane. (n=3, Mean±SD, ^**^*P*<0.01)


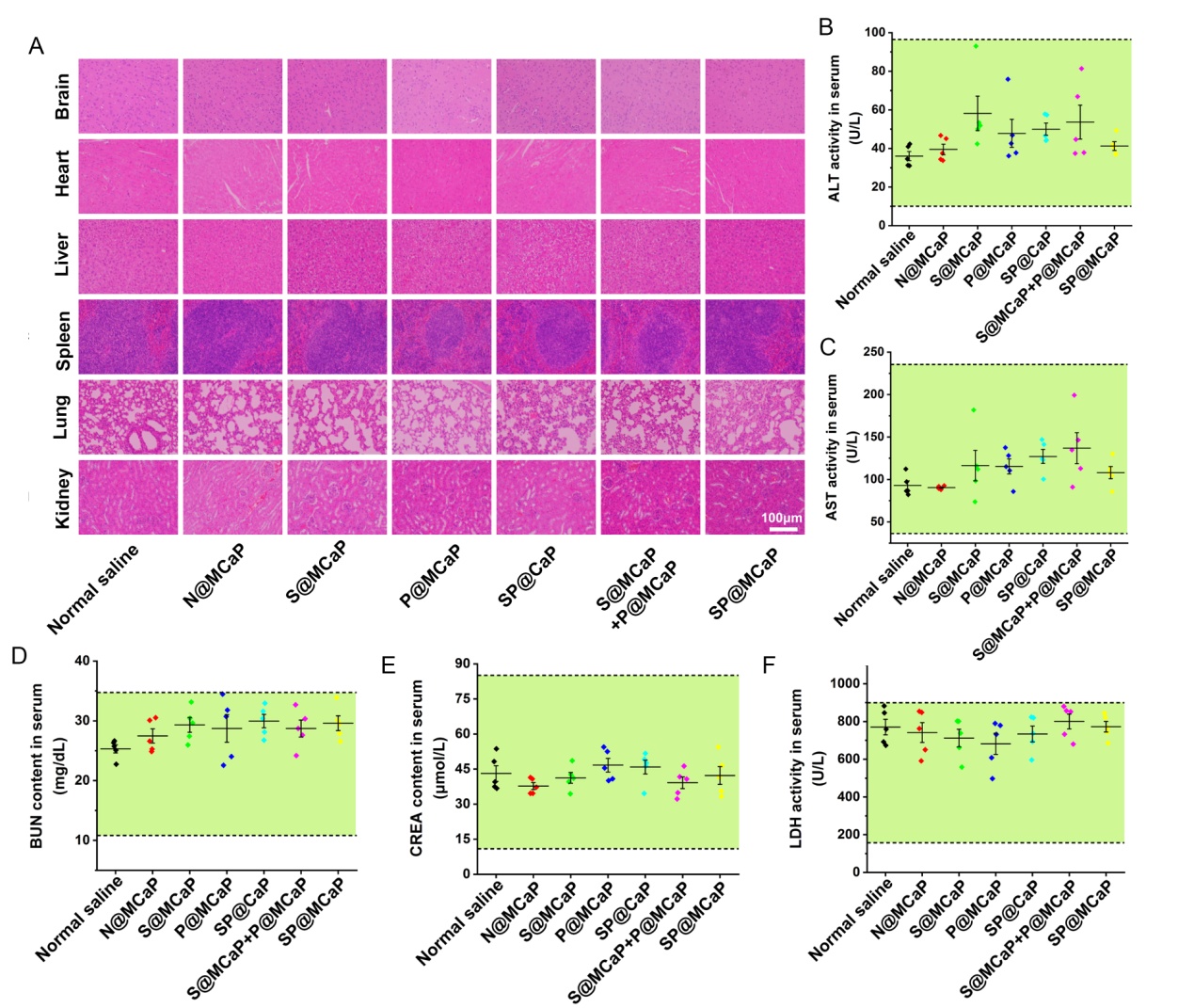


**Fig. S4.** Preliminary safety evaluation of SP@MCaP *in vivo*. (A) The typical picture of *H&E* staining of main organs in nude mice. (B-F) Effects of SP@MCaP on ALT activity, AST activity, BUN content, CREA content and LDH activity in serum of nude mice (green area indicates the normal range). (n=5, Mean±SD)


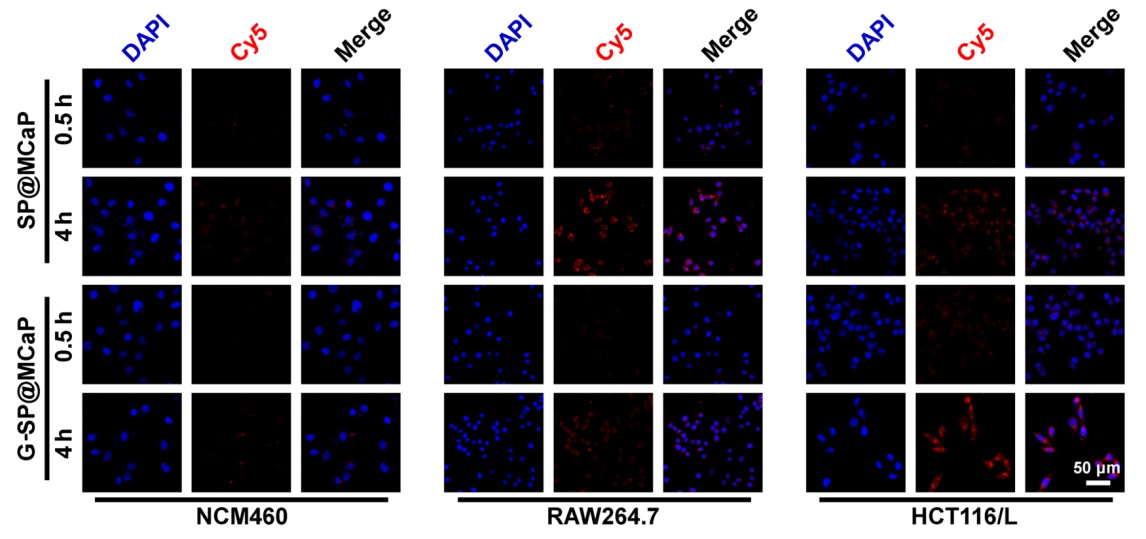


**Fig. S5.** The typical LSCM pictures of the uptake of G-SP@MCaP and SP@MCaP by co-cultured NCM460 cells, RAW264.7 cells and HCT116/L cells.


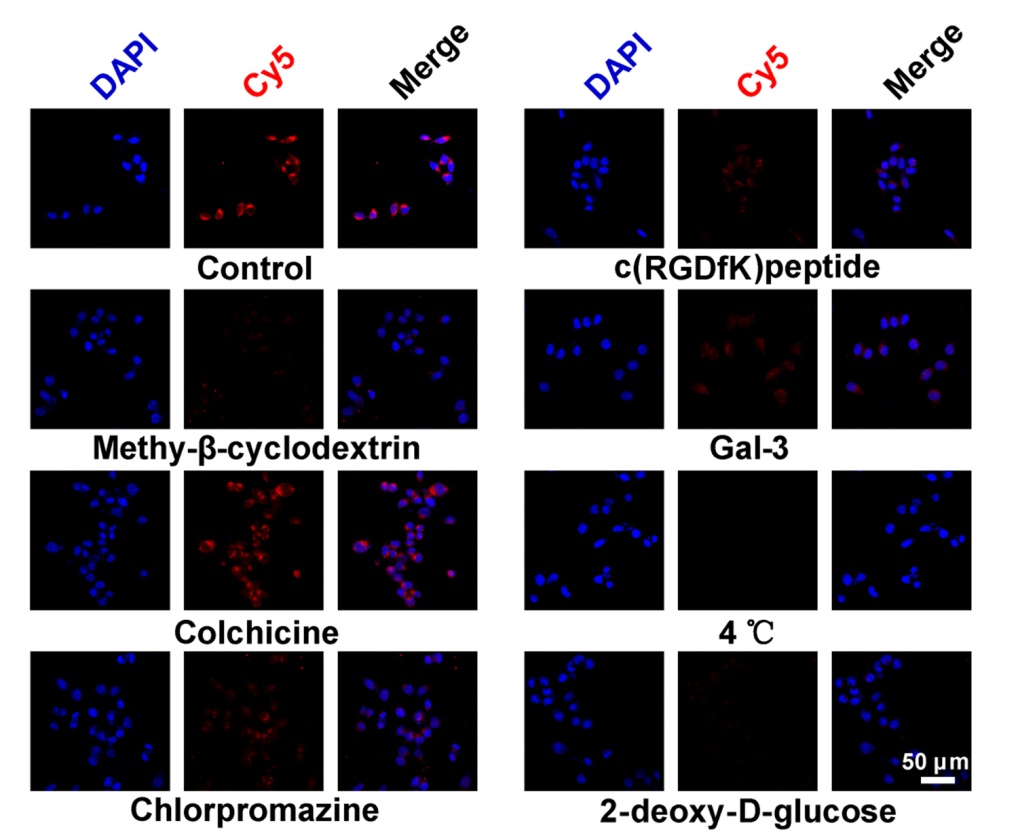


**Fig. S6.** The effects of different inhibitors on the uptake of G-SP@MCaP by HCT116/L cells.


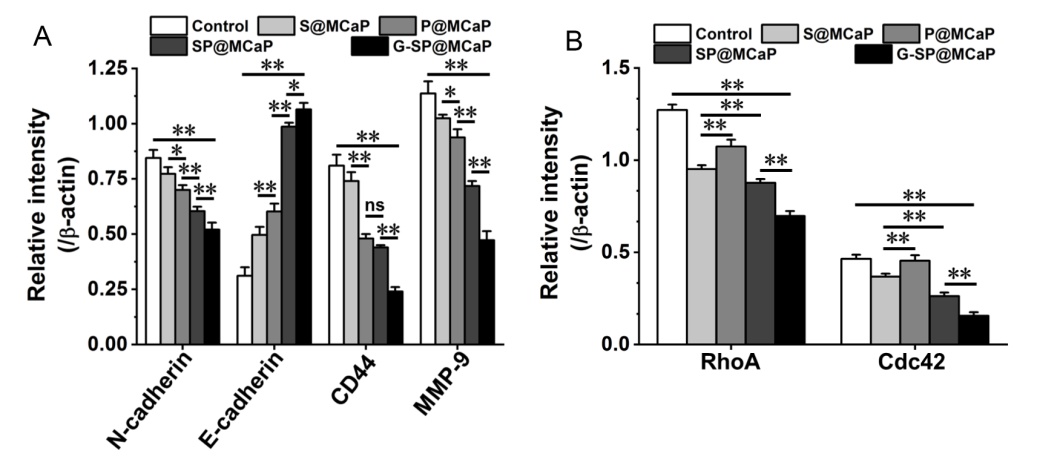


**Fig. S7.** The semi-quantitative analysis of invasion-related protein (A) and motion-related protein (B) in HCT116/L cells. (n=3, Mean±SD, ^*^*P*<0.05, ^**^*P*<0.01, ns: no significant difference)


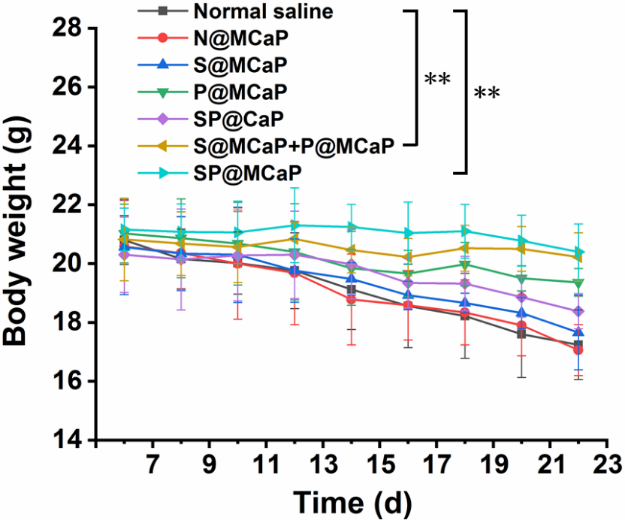


**Fig. S8.** The Effect of SP@MCaP on body weight of nude mice with orthotopic drug-resistant colon cancer. (n=5, Mean±SD, ^**^*P*<0.01)


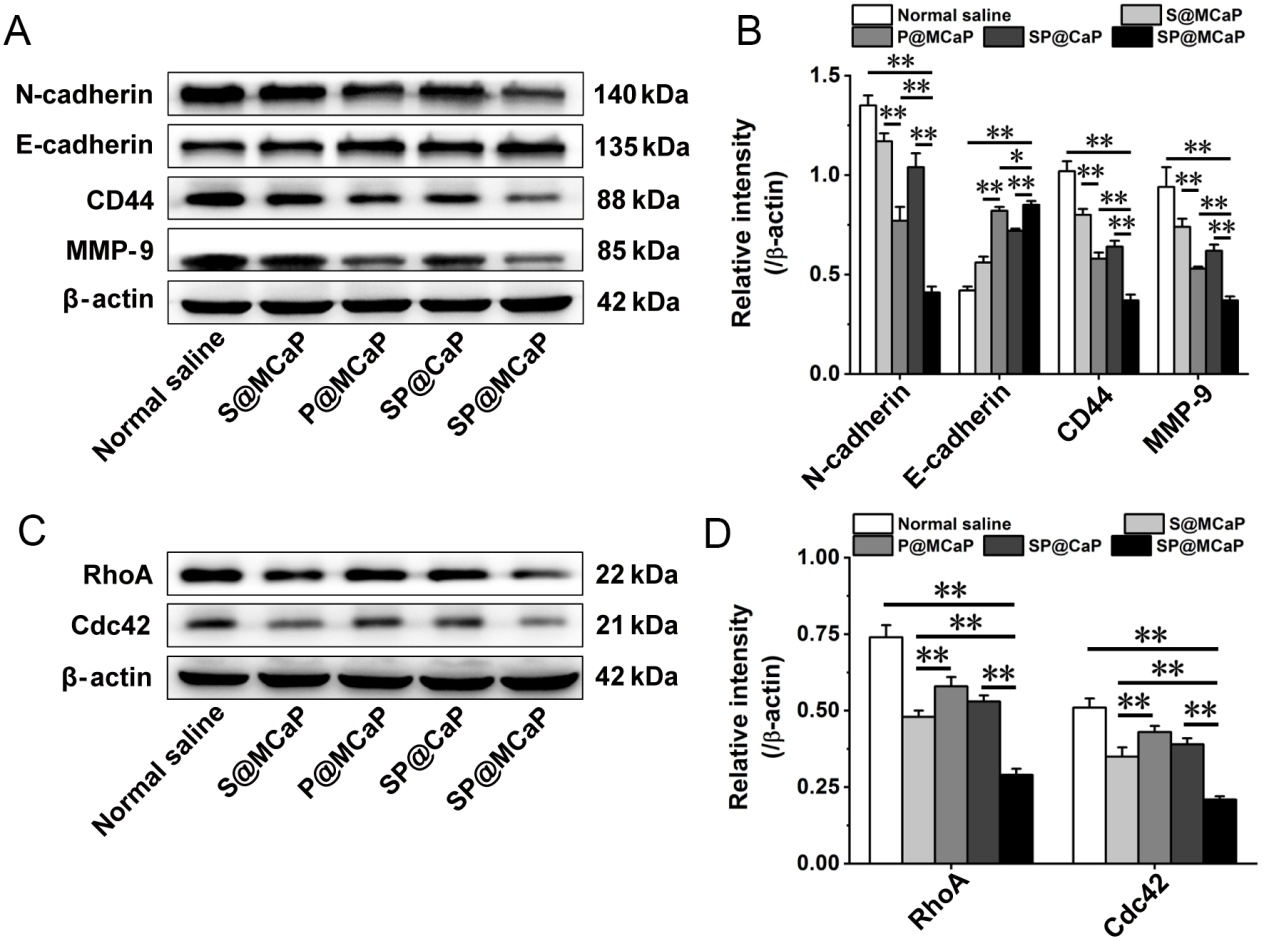


**Fig. S9.** The effects of SP@MCaP on invasion and motion-related protein expression in orthotopic drug-resistant colon cancer tissue. (A and B) Expression of invasion-related proteins. (C and D) Expression of motion-related proteins. (n=3, Mean±SD, ^*^*P*<0.05, ^**^*P*<0.01)


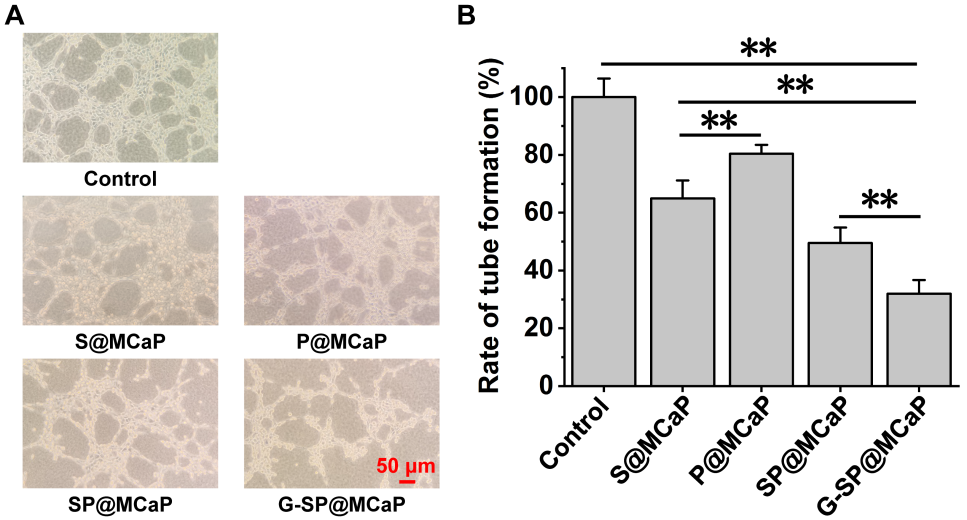


**Fig. S10.** The inhibitory effect of G-SP@MCaP on the tubule formation of HUVEC cells. (A) Typical pictures of tubule formation of HUVEC cells. (B) Statistical results of tubule formation of HUVEC cells. (n=3, Mean±SD, ^**^*P*<0.01)
